# Associations of sleep duration and sleep-timing patterns with depressive mood and suicidal outcomes in Korean adolescents

**DOI:** 10.1186/s12888-026-08706-6

**Published:** 2026-10-13

**Authors:** Heerim Son, Bong-Jo Kim, Boseok Cha, So-Jin Lee, Nuree Kang, Dongyun Lee, Eun Ji Lim, Soyoung Park, Jun Won Kim, Kyoung Min Kim, Jae-Won Choi

**Affiliations:** 1 Department of Psychiatry, Gyeongsang National University Hospital, 79 Gangnam-ro, Jinju, 52727 Republic of Korea; 2 Department of Psychiatry, Gyeongsang National University Changwon Hospital, Changwon, Republic of Korea; 3 Department of Psychiatry, Gyeongsang National University College of Medicine, Jinju, Republic of Korea; 4 Department of Psychiatry, Daegu Catholic University Medical Center, Daegu, Republic of Korea; 5 Department of Psychiatry, Daegu Catholic University School of Medicine, Daegu, Republic of Korea; 6 Department of Psychiatry, Dankook University Hospital, Cheonan, Republic of Korea; 7 Department of Psychiatry, College of Medicine, Dankook University, Cheonan, Republic of Korea

**Keywords:** Adolescent, Sleep duration, Sleep timing, MSFsc, Weekday–weekend sleep midpoint difference, Depressive mood, Suicidality, Korea youth risk behavior survey

## Abstract

**Background:**

Sleep duration, sleep timing, and weekday–weekend sleep-timing differences are interrelated characteristics associated with adolescent mental health. Categorizing continuous measures and interpreting mutually adjusted estimates as independent effects may obscure nonlinear patterns and their shared derivation. We examined associations of corrected mid-sleep on free days (MSFsc), weekly average sleep duration, and the absolute weekday–weekend sleep midpoint difference with depressive mood and suicidal outcomes.

**Methods:**

This cross-sectional study used data from the nationally representative 2025 Korea Youth Risk Behavior Survey. Descriptive analyses included 48,426 adolescents with valid sleep-derived variables, and adjusted models included 48,416 complete cases. Survey-weighted logistic regression with restricted cubic splines evaluated nonlinear associations of the three continuous sleep descriptors with depressive mood, suicidal ideation, suicide plan, and suicide attempt. Secondary analyses examined categorical sleep-timing groups, sequential adjustment models, and exploratory combined sleep-timing group–sleep-duration profiles.

**Results:**

Weekly average sleep duration and the absolute weekday–weekend sleep midpoint difference were associated with all four outcomes. Sleep duration was nonlinear for all outcomes, with higher odds at shorter durations. The midpoint difference was nonlinear only for depressive mood and was otherwise generally monotonic. MSFsc showed overall and nonlinear associations with depressive mood, suicidal ideation, and suicide plan, but not suicide attempt in the primary analysis (overall *p* = 0.054; nonlinearity *p* = 0.993); this result was sensitive to exclusion of extreme MSFsc values. Categorical sleep-timing group estimates changed substantially after adjustment for sleep duration and midpoint difference. In exploratory combined-profile analyses, < 5 h of sleep was associated with higher odds across all sleep-timing groups, but only 4 of 72 direct contrasts remained significant after false-discovery-rate adjustment.

**Conclusions:**

Very short sleep duration and larger weekday–weekend sleep midpoint differences showed the most consistent associations with depressive mood and suicidal outcomes. MSFsc associations varied across outcomes and were less robust for suicide attempt. Because the sleep descriptors share timing information, mutually adjusted estimates should be interpreted as conditional associations rather than independent causal effects. Longitudinal studies using more detailed circadian and sleep measures are needed.

**Supplementary Information:**

The online version contains supplementary material available at https://doi.org/10.1186/s12888-026-08706-6.

## Background

Adolescents’ mental health is a major public health concern. Globally, one in seven individuals aged 10–19 years experiences a mental disorder, and suicide is the third leading cause of death among those aged 15–29 years [1]. In Korea, the 2025 Korea Youth Risk Behavior Survey (KYRBS), a nationally representative school-based survey of Korean middle- and high-school students, reported that 25.7% of students experienced depressive mood and 11.6% reported suicidal ideation during the past 12 months [2]. Meta-analytic evidence further indicates that depressive symptoms are prevalent during adolescence and may show continuity over time, including among Korean youth [3, 4]. Identifying modifiable behavioral factors associated with depressive mood and suicidal outcomes is therefore an important public health priority.

Sleep is one such potentially modifiable factor. A systematic review and meta-analysis of 74 studies found that shorter sleep duration was associated with poorer mood-related outcomes, including depressive symptoms, anxiety, and negative affect [5]. Prospective evidence also indicates that broadly defined sleep disturbances are associated with the subsequent onset of mood or psychotic disorders during adolescence and early adulthood [6]. Sleep problems have been associated with emotion-regulation difficulties in children and adolescents [7] and with suicidal ideation, suicide plans, and suicide attempts, although prospective evidence has been more consistent for suicidal ideation than for suicide attempts [8]. Sleep may be relevant to these outcomes because it supports cognitive and emotional functioning during a period of substantial brain maturation [9–12]. Insufficient sleep has also been associated with greater risk-taking among adolescents, providing another potential pathway linking sleep to suicidal behavior [13].

Adolescent sleep is shaped by both developmental and social influences. Sleep timing generally shifts later during adolescence and begins to advance again after late adolescence [14, 15]. This developmental shift may conflict with fixed early school schedules and other social demands, contributing to insufficient sleep on school days and differences in sleep timing between school days and free days [16, 17]. These weekday–weekend differences have commonly been described as social jetlag, reflecting a discrepancy between biological and socially required timing [17]. Meta-analytic evidence suggests that later school start times are associated with later sleep timing, longer weekday sleep duration, and smaller weekday–weekend timing differences among adolescents [18]. In this context, the American Academy of Sleep Medicine recommends that adolescents aged 13–18 years obtain 8–10 h of sleep per 24 h on a regular basis [19]. Sleep duration, sleep timing, and weekday–weekend timing differences may therefore co-occur but represent non-equivalent dimensions of adolescent sleep.

These structural constraints may be particularly relevant in Korea. Regional evaluations of delayed school start policies implemented in 2014–2015 reported shifts toward later school start times and modest increases in adolescent sleep duration, although the magnitude and persistence of these changes varied across studies [20, 21]. Korean students may also face substantial educational demands outside regular school hours. In the 2025 national survey of private-education expenditures, 73.0% of middle-school students and 63.0% of high-school students participated in private education, with reported weekly participation times of 7.2 and 6.6 h, respectively [22]. These educational demands may constrain sleep opportunities, while academic stress may also be associated with mental health beyond the potential effects of academic demands on sleep.

Previous studies have linked each of the major sleep dimensions examined in this study to adolescent mental health, but several interpretive uncertainties remain. A dose–response meta-analysis found that the associations between sleep duration and suicidality differed across suicidal outcomes and included both linear and nonlinear patterns [23]. Eveningness has also been associated with greater depressive symptoms, although the magnitude of the association varies according to the chronotype measure used [24]. In Korean adolescents, weekday sleep timing showed a J-shaped association with suicidal ideation, with higher odds at both relatively early and late sleep times [25]. Evidence concerning weekday–weekend sleep-timing differences has been less consistent. An earlier systematic review found heterogeneous and equivocal evidence regarding their associations with mental health in young people [26], whereas a more recent meta-analysis found that social jetlag of at least 2 h was associated with higher odds of depressive symptoms, albeit with substantial between-study heterogeneity [27]. A study of Japanese adolescents further reported nonlinear and sex-specific associations between social jetlag and depressive symptoms [28]. Together, these findings indicate that results may depend on how sleep timing is measured and modeled and that categorization may obscure nonlinear or threshold patterns.

An additional methodological challenge is that sleep duration, sleep timing, and weekday–weekend timing differences are derived from overlapping sleep-timing information. In the present study, corrected mid-sleep on free days (MSFsc) was therefore treated as a behavioral sleep-timing proxy rather than as a direct measure of trait-like circadian preference. Because MSFsc, average weekly sleep duration, and the absolute weekday–weekend sleep midpoint difference share component variables, mutually adjusted estimates should be interpreted as conditional associations rather than as independent causal effects or evidence of mediation. To address these methodological issues, we used data from the 2025 KYRBS to examine the survey-weighted, potentially nonlinear associations of continuous MSFsc, average weekly sleep duration, and the absolute weekday–weekend sleep midpoint difference with depressive mood, suicidal ideation, suicide plan, and suicide attempt among Korean middle- and high-school students. Secondary analyses evaluated categorical MSFsc-based sleep-timing groups and their combined profiles with sleep-duration categories. Prespecified and post hoc sensitivity analyses were conducted to assess the robustness of the findings.

## Methods

### Study design and data source

This cross-sectional study analyzed data from the 21st Korea Youth Risk Behavior Survey (KYRBS), conducted in 2025 by the Korea Disease Control and Prevention Agency (KDCA) [2]. The KYRBS is a nationally representative, school-based survey of Korean middle- and high-school students that uses a complex sampling design. The public-use dataset includes the sampling weights and design variables required for design-based analyses, including stratum identifiers, cluster identifiers, and finite population correction variables [29].

### Study population

**Fig. 1:**
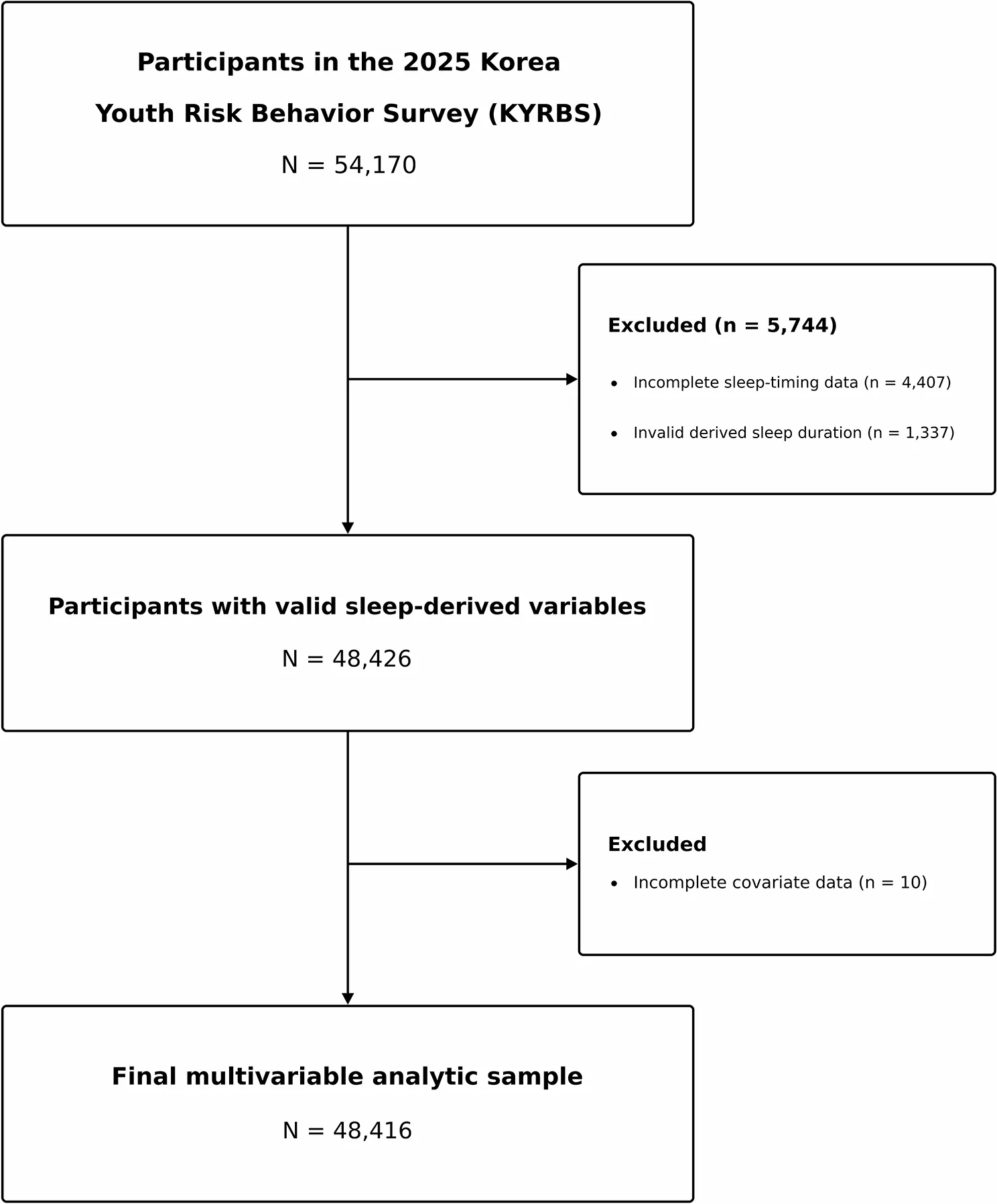
Participant flow through the descriptive and multivariable analytic samples. Among 54,170 participants in the 2025 Korea Youth Risk Behavior Survey, 4,407 were excluded because of incomplete sleep-timing information and 1,337 because of implausible derived sleep-duration values. The resulting 48,426 participants with valid sleep-derived variables were included in the descriptive analyses. A further 10 participants with incomplete data for variables required in the adjusted models were excluded, leaving 48,416 participants in the final multivariable analytic sample

Eligible participants were students enrolled in middle school grade 1 through high school grade 3. The observed age range was 12–18 years. Among the 54,170 students who participated in the 2025 survey, 48,426 met the prespecified validity criteria for sleep-timing and derived sleep variables, including exclusion of derived sleep durations ≤ 0 or ≥ 24 h, and were included in the descriptive analyses. An additional 10 students with incomplete data for variables required in the adjusted models were excluded, leaving 48,416 students for multivariable analyses. The sequential participant exclusions are presented in Fig. 1. Survey-weighted comparisons of participants included in and excluded from the valid sleep-derived sample are presented in Supplementary Table S1.

### Sleep timing, sleep duration, and weekday–weekend sleep midpoint difference

Sleep timing was derived from KYRBS items asking participants to report their usual bedtime and wake time separately for weekdays and weekends during the past seven days. Values coded as 9999 were treated as missing. The public-use data had undergone the official KDCA data-cleaning procedures. In accordance with the KDCA processing rules, weekday wake times earlier than 04:00 or later than 09:00 were treated as missing [29]. Because the KDCA did not specify an official exclusion rule for weekend bedtime, responses between 04:00 and 19:59 were retained in the primary analyses to avoid imposing a post hoc exclusion criterion. Their influence was evaluated in a sensitivity analysis that excluded these responses. Derived sleep durations of ≤ 0 h or ≥ 24 h were considered invalid and treated as missing.

To account for sleep periods crossing midnight, bedtime and wake time were converted to an extended clock-time scale before calculating sleep duration and sleep midpoints, following the algorithm provided in the KDCA syntax [29]. Sleep duration was calculated separately for weekdays and weekends as the interval between bedtime and wake time. Average weekly sleep duration was calculated as follows:

SDweek = (5 × weekday sleep duration + 2 × weekend sleep duration) / 7

and was converted to hours for analysis.

For the categorical secondary analyses, average weekly sleep duration was grouped into six categories: <5, 5 to < 6, 6 to < 7, 7 to < 8, 8 to < 9, and ≥ 9 h.

The midpoint of sleep was calculated separately for weekdays (MSW) and weekends (MSF). The primary measure was the absolute weekday–weekend sleep midpoint difference, calculated as |MSF − MSW| / 60, which represented the magnitude of the sleep-timing discrepancy irrespective of direction [17]. Because this measure did not directly assess circadian phase and the term “social jetlag” may imply a physiological phase shift, we use the descriptive term “weekday–weekend sleep midpoint difference” [30, 31]. In a sensitivity analysis, we additionally examined the signed difference, calculated as (MSF − MSW) / 60; positive values indicated a later weekend midpoint and negative values an earlier weekend midpoint.

### MSFsc assessment and categorical sleep-timing groups

The corrected midpoint of sleep on free days (MSFsc) was used as a behavioral sleep-timing proxy. MSFsc was derived from the Munich ChronoType Questionnaire framework and corrects the midpoint of sleep on free days for oversleep attributable to accumulated sleep debt [32, 33]. In the present study, weekends were operationalized as free days. When weekend sleep duration did not exceed weekday sleep duration, MSFsc was set equal to MSF. When weekend sleep duration exceeded weekday sleep duration, MSFsc was calculated as follows [32, 33]:

MSFsc = MSF − [(weekend sleep duration − average weekly sleep duration) / 2].

MSFsc was expressed on the unwrapped extended-hour scale used in the KDCA processing syntax to preserve the temporal ordering of sleep midpoints across midnight [29].

MSFsc was retained as a continuous variable in the primary nonlinear analyses. For secondary categorical analyses, participants were classified into morning, intermediate, and evening MSFsc-based sleep-timing groups using the unweighted 20th and 80th percentiles of the MSFsc distribution among the 48,426 participants with valid sleep-derived variables. This distribution-based approach was consistent with previous Korean adolescent research using MSFsc-derived categories [34]. The locked cutoffs were 27.7619 and 29.9524 h on the extended scale, corresponding to approximately 03:46 and 05:57, respectively. Participants with MSFsc values below the 20th percentile were classified into the morning group; those with values from the 20th through the 80th percentile, including both boundary values, into the intermediate group; and those with values above the 80th percentile into the evening group. The distributional summary, percentile thresholds, and classification rules are presented in Supplementary Table S2, and the weighted MSFsc distribution with the locked classification thresholds is shown in Supplementary Figure S1. Because the KYRBS did not administer the full Munich ChronoType Questionnaire or a morningness–eveningness preference questionnaire, MSFsc and the percentile-based categories were interpreted as sleep-timing-derived measures and not as direct assessments of trait-like chronotype.

Extreme extended-scale MSFsc values below 24 h or above 36 h were retained in the primary analyses. To evaluate their influence, analyses excluding these 514 observations were conducted as sensitivity analyses. Additional sensitivity analyses applied survey-weighted 20th and 80th percentile cutoffs and survey-weighted 25th and 75th percentile cutoffs.

### Mental health outcomes

Four binary mental health outcomes were examined: depressive mood, suicidal ideation, suicide plan, and suicide attempt. All outcomes referred to the past 12 months and were assessed using the corresponding standardized KYRBS items [2]. Depressive mood was defined as having experienced sadness or hopelessness for at least two consecutive weeks to the extent that usual activities were disrupted. Suicidal ideation, suicide plan, and suicide attempt were defined as having seriously considered suicide, making a specific suicide plan, or attempting suicide, respectively. Each outcome was coded as 1 for “yes” and 0 for “no.”

### Covariates

The primary sociodemographic covariates were sex, school grade, socioeconomic status, and academic performance. The adjustment set was specified before the reviewer-requested reanalyses and was limited to broad sociodemographic and educational characteristics plausibly associated with both adolescent sleep patterns and mental health. We avoided extensive adjustment for psychological and behavioral variables with uncertain temporal ordering that could lie on the causal pathway. Sex was recorded as male or female based on a self-administered KYRBS survey item. School grade was coded ordinally from 1 to 6, corresponding to middle school grades 1–3 and high school grades 1–3, and was entered as a linear term in the adjusted models. The six grade levels were displayed separately in descriptive analyses. Because grade coding identified middle- versus high-school stage, a separate broad school-level indicator was not included in the adjusted models. Socioeconomic status and academic performance were each entered as five-level categorical variables, with the highest socioeconomic status and best academic performance categories used as the respective reference groups. Sleep duration, MSFsc, and the weekday–weekend sleep midpoint difference were treated as the main sleep-related variables rather than as sociodemographic covariates. Because these measures were derived from overlapping sleep-timing information, they were considered interrelated descriptors of the same sleep pattern rather than causally independent exposures.

In a sensitivity analysis, weekly average academic-purpose sitting time was additionally included. Academic-purpose sitting time was derived from weekday and weekend time spent sitting for school or private-academy classes, homework, or studying and was used as a broad proxy for academic load. Individual school start and end times and private-academy attendance duration were not separately available in the KYRBS.

### Statistical analysis

All analyses accounted for the complex sampling design of the KYRBS, including sampling weights, strata, clusters, and finite population correction variables. Analyses were conducted using IBM SPSS Statistics version 27 and R version 4.6.1 with the survey package version 4.5. Descriptive analyses included 48,426 participants with valid sleep-derived variables, whereas adjusted models used the final complete-case sample of 48,416 participants.

Categorical variables were summarized as unweighted counts and survey-weighted percentages, and continuous variables as survey-weighted means with standard errors. Differences across MSFsc-based sleep-timing groups were assessed using Rao–Scott adjusted chi-square tests for categorical variables and design-based Wald *F*-tests for continuous variables. Survey-weighted comparisons were also conducted between participants included in and excluded from the valid sleep-derived sample. Survey-weighted Pearson correlation coefficients and design-adjusted *p* values were calculated among MSFsc, average weekly sleep duration, and the absolute weekday–weekend sleep midpoint difference. Collinearity was evaluated using tolerance and variance inflation factors obtained from sampling-weighted auxiliary linear regressions. These diagnostics were used to assess variance inflation but do not eliminate the mathematical coupling inherent in the derivation of the sleep descriptors.

To avoid information loss from categorization and to assess departures from linearity, the primary analyses retained MSFsc, average weekly sleep duration, and the absolute weekday–weekend sleep midpoint difference as continuous exposures and modeled each using survey-weighted logistic regression with restricted cubic spline terms [35]. Separate models were fitted for depressive mood, suicidal ideation, suicide plan, and suicide attempt. Four knots were used to allow flexible modeling of nonlinear associations while limiting model complexity across the exposure–outcome models. Following recommended quantile locations for a four-knot restricted cubic spline, knots were placed at the survey-weighted 5th, 35th, 65th, and 95th percentiles of each exposure [36]. The survey-weighted median was used as the reference value to provide a central comparison point within the observed distribution. When the focal exposure was modeled with spline terms, the other two sleep descriptors were included as linear terms. All models were adjusted for sex, school grade, five-level socioeconomic status, and five-level academic performance. Overall associations were assessed using joint design-based Wald tests of all three spline terms, and nonlinearity was assessed using joint Wald tests of the two nonlinear spline terms. Odds ratios and 95% confidence intervals were calculated over prediction grids spanning the survey-weighted 1st to 99th percentiles; observations outside this plotting range remained included in model fitting.

Secondary categorical analyses were retained for interpretability and continuity with the originally submitted analysis and were not used in place of the primary continuous spline analyses. These analyses examined morning, intermediate, and evening MSFsc-based sleep-timing groups. Crude models were followed by mutually adjusted models that included average weekly sleep duration, the absolute weekday–weekend sleep midpoint difference, and the sociodemographic covariates. Sequential models were also fitted to examine changes in the sleep-timing group estimates across adjustment sets. Model A adjusted for sex, school grade, socioeconomic status, and academic performance; Model B additionally included average weekly sleep duration; Model C additionally included the absolute weekday–weekend sleep midpoint difference; and Model D included both average weekly sleep duration and the absolute midpoint difference. Changes across these models were interpreted as conditional associations and not as evidence of mediation.

Combined sleep-timing group–sleep duration profiles were examined using an 18-level categorical variable formed by crossing the three MSFsc-based sleep-timing groups with the six sleep-duration categories. This secondary exploratory analysis was intended to describe outcome odds across clinically interpretable combinations of sleep timing and sleep duration that are not displayed by a conventional main-effects model; it was not used as primary evidence of interaction or effect modification. These models were adjusted for sex, school grade, socioeconomic status, academic performance, and the absolute weekday–weekend sleep midpoint difference. The morning group with 8 to < 9 h of sleep was used as the reference profile. The multiplicative interaction between sleep-timing group and sleep duration was assessed using a design-adjusted omnibus Wald *F*-test. Direct pairwise contrasts among the three sleep-timing groups were additionally estimated within each sleep-duration category. Because these combined-profile analyses were exploratory, the Benjamini–Hochberg false discovery rate procedure was applied across all 72 direct within-stratum contrasts, and both raw *p* values and false discovery rate-adjusted *q* values were reported.

Sensitivity analyses included: (1) replacing the absolute midpoint difference with the signed weekend-minus-weekday midpoint difference; (2) excluding 4,465 participants with weekend bedtime responses between 04:00 and 19:59, leaving 43,951 participants; (3) restricting both weekday and weekend sleep duration to 3–14 h, leaving 46,993 participants; (4) replacing average weekly sleep duration with weekday sleep duration or weekend sleep duration in separate models and, in a third specification, jointly including weekday sleep duration and weekend sleep extension, defined as weekend sleep duration minus weekday sleep duration; (5) additional adjustment for weekly average academic-purpose sitting time, modeled using restricted cubic splines among 47,565 participants; (6) alternative MSFsc classifications using survey-weighted 20th and 80th percentile cutoffs and survey-weighted 25th and 75th percentile cutoffs; and (7) a post hoc analysis excluding 514 observations with extreme extended-scale MSFsc values below 24 or above 36 h, leaving 47,902 participants. The extreme-value models retained the same knots, reference values, covariates, and prediction grids as the primary spline models. Supplementary Tables S3–S9 present, respectively, the correlation and collinearity diagnostics, sequential models, summary sensitivity analyses, detailed sleep-timing sensitivity analyses, weekday and weekend sleep-duration models, direct within-stratum contrasts, and pooled joint-profile estimates.

All tests were two-sided, and statistical significance was defined as *p* < 0.05. Because the four mental health outcomes were correlated but clinically distinct prespecified outcomes and the primary analyses comprised a limited prespecified set of exposure–outcome models, *p* values for the primary analyses were not multiplicity-adjusted. Given the large sample size, primary findings were interpreted with emphasis on effect estimates, 95% confidence intervals, and consistency across outcomes rather than isolated *p* values. Multiplicity adjustment was reserved for the 72 exploratory direct within-stratum contrasts, for which Benjamini–Hochberg false-discovery-rate-adjusted *q* values were reported.

### Ethical considerations

This study used publicly available, de-identified secondary data from the 2025 KYRBS conducted by the KDCA. The KYRBS is a government-approved national survey, and participation was voluntary and anonymous according to the survey protocol. This study was conducted in accordance with the principles of the Declaration of Helsinki. The present secondary analysis was reviewed and determined to be exempt by the Institutional Review Board of Gyeongsang National University Hospital (approval no. GNUH 2026-06-001).

## Results

### Participant flow and analytic samples

Of the 54,170 students who participated in the 2025 KYRBS, 48,426 met the prespecified validity criteria for sleep-timing and derived sleep variables and were included in the descriptive analyses. A further 10 participants with incomplete data for variables required in the adjusted models were excluded, leaving 48,416 participants in the final complete-case analytic sample (Fig. 1). Survey-weighted comparisons between participants included in and excluded from the valid sleep-derived sample are presented in Supplementary Table S1. Between-group differences in sociodemographic characteristics were generally small to modest; however, excluded participants had higher prevalences of depressive mood, suicidal ideation, suicide plan, and suicide attempt than included participants (Supplementary Table S1).

### Participant characteristics and sleep patterns

Among the 48,426 participants included in the descriptive analyses, 9,643 (19.9%) were classified into the morning group, 29,123 (60.1%) into the intermediate group, and 9,660 (19.9%) into the evening group. The survey-weighted prevalences of depressive mood, suicidal ideation, suicide plan, and suicide attempt were 25.2%, 11.2%, 4.1%, and 2.3%, respectively (Supplementary Table S1). The distribution of MSFsc-based sleep-timing groups differed according to sex, school grade, socioeconomic status, and academic performance (all *p* < 0.001; Table 1). Sleep timing and duration also differed across the groups (all *p* < 0.001; Table 1). Sleep timing shifted progressively later from the morning to the intermediate and evening groups on both weekdays and weekends. Compared with the morning group, the evening group had shorter weekday sleep duration (5.72 vs. 6.97 h), shorter weekly average sleep duration (6.45 vs. 7.55 h), and a larger absolute weekday–weekend sleep midpoint difference (3.42 vs. 0.92 h). The survey-weighted distributions of weekly average sleep duration and the absolute weekday–weekend sleep midpoint difference are shown in Supplementary Figure S2; substantial overlap remained across the three groups.

**Table 1:** Sociodemographic characteristics and sleep patterns by MSFsc-based sleep-timing group

Characteristic	Category	Total (*N* = 48,426)	Morning (*n* = 9,643)	Intermediate (*n* = 29,123)	Evening (*n* = 9,660)	*p* value
Sex	Male	24,619	5,332 (21.9)	14,424 (58.4)	4,863 (19.7)	< 0.001
	Female	23,807	4,311 (18.1)	14,699 (61.6)	4,797 (20.3)	
Grade	Middle school grade 1	8,484	2,727 (32.8)	4,552 (53.3)	1,205 (14.0)	< 0.001
	Middle school grade 2	8,346	1,863 (22.4)	4,810 (57.6)	1,673 (20.0)	
	Middle school grade 3	8,228	1,463 (17.7)	4,844 (58.4)	1,921 (23.9)	
	High school grade 1	7,957	1,322 (16.7)	4,994 (62.5)	1,641 (20.8)	
	High school grade 2	7,726	1,108 (14.7)	4,927 (63.4)	1,691 (22.0)	
	High school grade 3	7,685	1,160 (15.1)	4,996 (65.3)	1,529 (19.6)	
Socioeconomic status	Level 1 (high)	5,113	1,222 (23.8)	3,041 (59.5)	850 (16.7)	< 0.001
	Level 2	15,301	3,095 (20.6)	9,483 (61.6)	2,723 (17.8)	
	Level 3	22,703	4,350 (18.9)	13,638 (60.1)	4,715 (21.0)	
	Level 4	4,474	817 (18.6)	2,511 (55.8)	1,146 (25.6)	
	Level 5 (low)	825	157 (19.2)	445 (53.8)	223 (26.9)	
Academic performance	Level 1 (high)	5,788	1,495 (26.2)	3,492 (60.0)	801 (13.8)	< 0.001
	Level 2	12,567	2,702 (21.7)	7,913 (62.7)	1,952 (15.6)	
	Level 3	14,061	2,696 (19.1)	8,768 (62.4)	2,597 (18.5)	
	Level 4	11,499	2,006 (17.6)	6,638 (57.3)	2,855 (25.1)	
	Level 5 (low)	4,506	743 (15.8)	2,309 (51.4)	1,454 (32.8)	
Sleep timing and duration	Weekday bedtime	00:50 (0.8 min)	23:46 (1.1 min)	00:55 (0.8 min)	01:34 (1.0 min)	< 0.001
	Weekday wake time	07:03 (0.5 min)	06:44 (0.6 min)	07:04 (0.5 min)	07:17 (0.6 min)	< 0.001
	Weekend bedtime	01:29 (0.7 min)	23:00 (1.5 min)	01:32 (0.5 min)	03:42 (0.9 min)	< 0.001
	Weekend wake time	09:47 (0.9 min)	08:01 (1.2 min)	09:38 (0.8 min)	11:59 (1.6 min)	< 0.001
	Weekday sleep duration, h	6.22 (0.01)	6.97 (0.02)	6.15 (0.02)	5.72 (0.02)	< 0.001
	Weekend sleep duration, h	8.31 (0.02)	9.02 (0.03)	8.10 (0.02)	8.28 (0.03)	< 0.001
	Weekly average sleep duration, h	6.82 (0.01)	7.55 (0.02)	6.71 (0.01)	6.45 (0.02)	< 0.001
	Absolute weekday–weekend sleep midpoint difference, h	1.83 (0.01)	0.92 (0.01)	1.59 (0.01)	3.42 (0.02)	< 0.001

Values are unweighted *n* (survey-weighted row %) for categorical variables and survey-weighted mean (SE) for continuous variables; SEs for bedtimes and wake times are expressed in minutes. Row percentages indicate the distribution of MSFsc-based sleep-timing groups within each characteristic category. *P* values were obtained using Rao–Scott adjusted chi-square tests for categorical variables and design-based Wald *F* tests for continuous variables. Socioeconomic status and academic performance were missing for 10 and 5 participants, respectively; available-case analyses were used. MSFsc, corrected midpoint of sleep on free days

### Correlations and collinearity among sleep descriptors

The three continuous sleep descriptors were modestly intercorrelated (Supplementary Table S3). MSFsc was positively correlated with the absolute weekday–weekend sleep midpoint difference (survey-weighted *r* = 0.49, *p* < 0.001) and inversely correlated with average weekly sleep duration (*r* = − 0.29, *p* < 0.001), whereas average weekly sleep duration and the absolute midpoint difference were only weakly correlated (*r* = 0.16, *p* < 0.001). In sampling-weighted auxiliary regressions, variance inflation factors were 1.594 for MSFsc, 1.501 for the absolute midpoint difference, and 1.250 for average weekly sleep duration (all tolerances > 0.62), indicating limited conventional variance inflation among the mutually adjusted sleep descriptors.

### Associations of continuous sleep descriptors with mental health outcomes

**Fig. 2:**
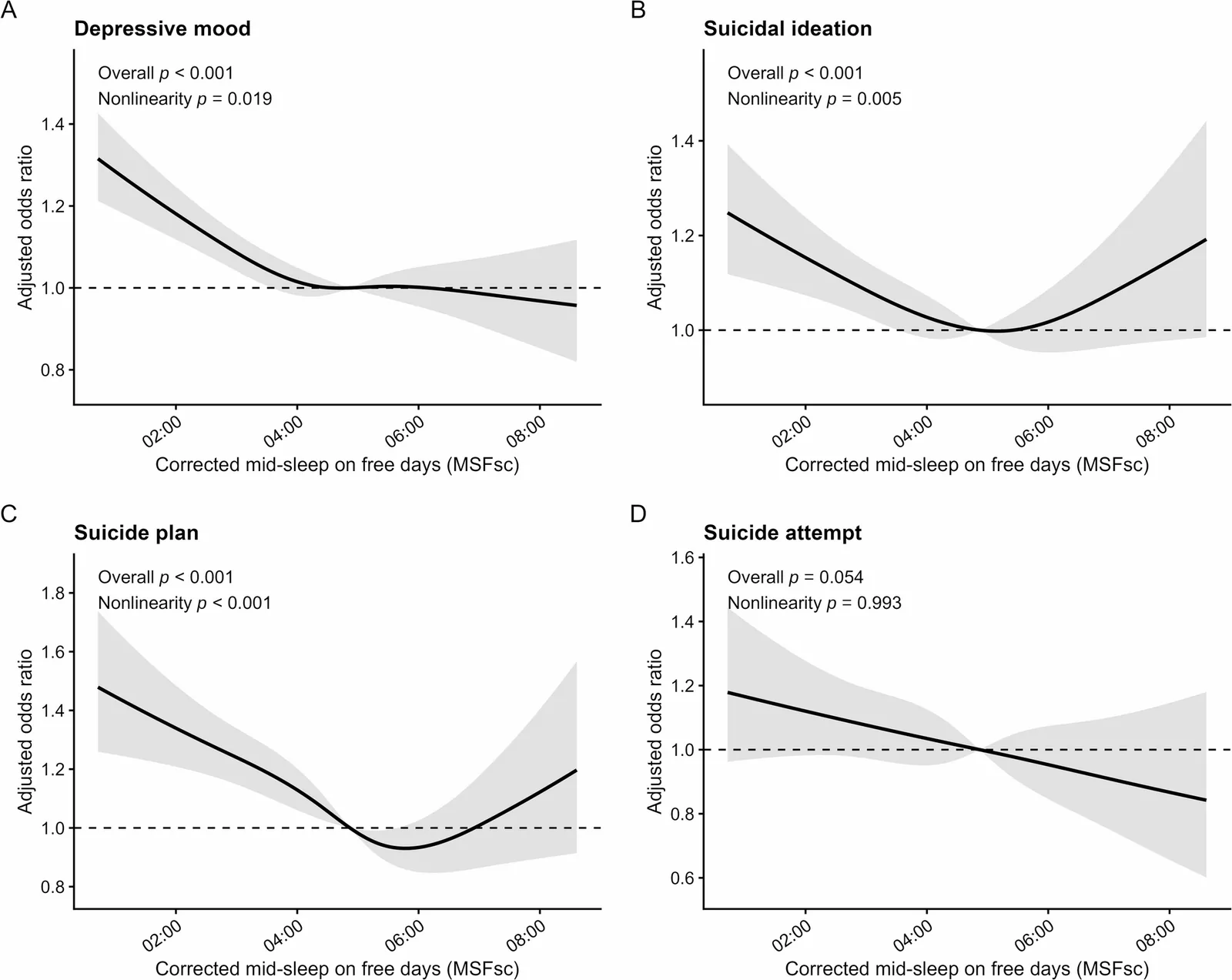
Adjusted associations of corrected mid-sleep on free days with depressive mood and suicidal outcomes. Survey-weighted restricted cubic spline curves show the associations of continuous corrected mid-sleep on free days (MSFsc) with (**A**) depressive mood, (**B**) suicidal ideation, (**C**) suicide plan, and (**D**) suicide attempt. Solid lines represent adjusted odds ratios, shaded areas represent 95% confidence intervals, and horizontal dashed lines indicate an odds ratio of 1. Four knots were placed at the survey-weighted 5th, 35th, 65th, and 95th percentiles of MSFsc, and the survey-weighted median was used as the reference value. Models were adjusted for sex, school grade, categorical socioeconomic status, categorical academic performance, weekly average sleep duration, and the absolute weekday–weekend sleep midpoint difference; the latter two sleep descriptors were entered as linear terms. Curves span the survey-weighted 1st to 99th percentiles, while all eligible observations remained included in model fitting. The analytic sample included 48,416 participants

**Fig. 3:**
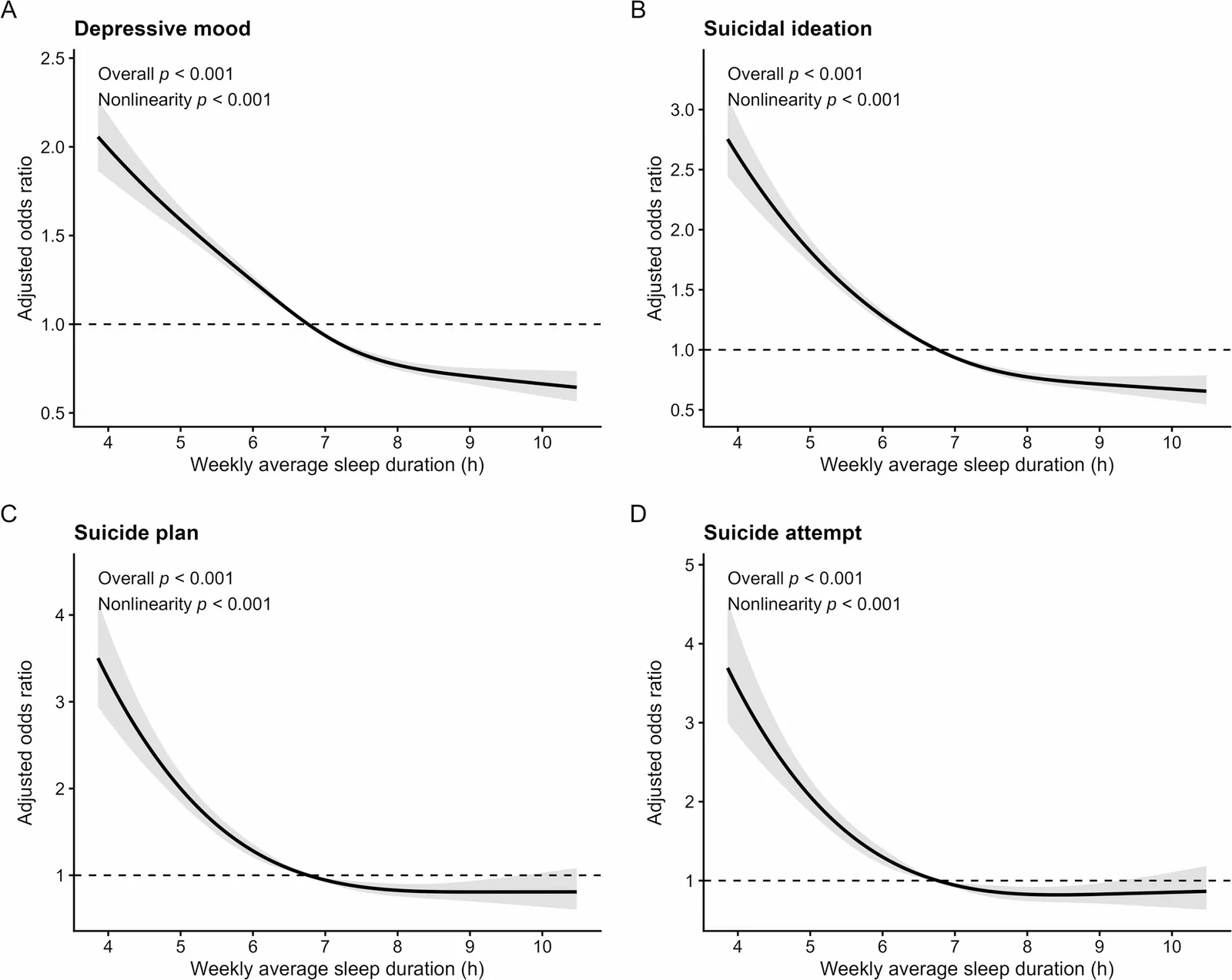
Adjusted associations of weekly average sleep duration with depressive mood and suicidal outcomes. Survey-weighted restricted cubic spline curves show the associations of continuous weekly average sleep duration with (**A**) depressive mood, (**B**) suicidal ideation, (**C**) suicide plan, and (**D**) suicide attempt. Solid lines represent adjusted odds ratios, shaded areas represent 95% confidence intervals, and horizontal dashed lines indicate an odds ratio of 1. Four knots were placed at the survey-weighted 5th, 35th, 65th, and 95th percentiles of weekly average sleep duration, and the survey-weighted median was used as the reference value. Models were adjusted for sex, school grade, categorical socioeconomic status, categorical academic performance, MSFsc, and the absolute weekday–weekend sleep midpoint difference; the latter two sleep descriptors were entered as linear terms. Curves span the survey-weighted 1st to 99th percentiles, while all eligible observations remained included in model fitting. The analytic sample included 48,416 participants

**Fig. 4:**
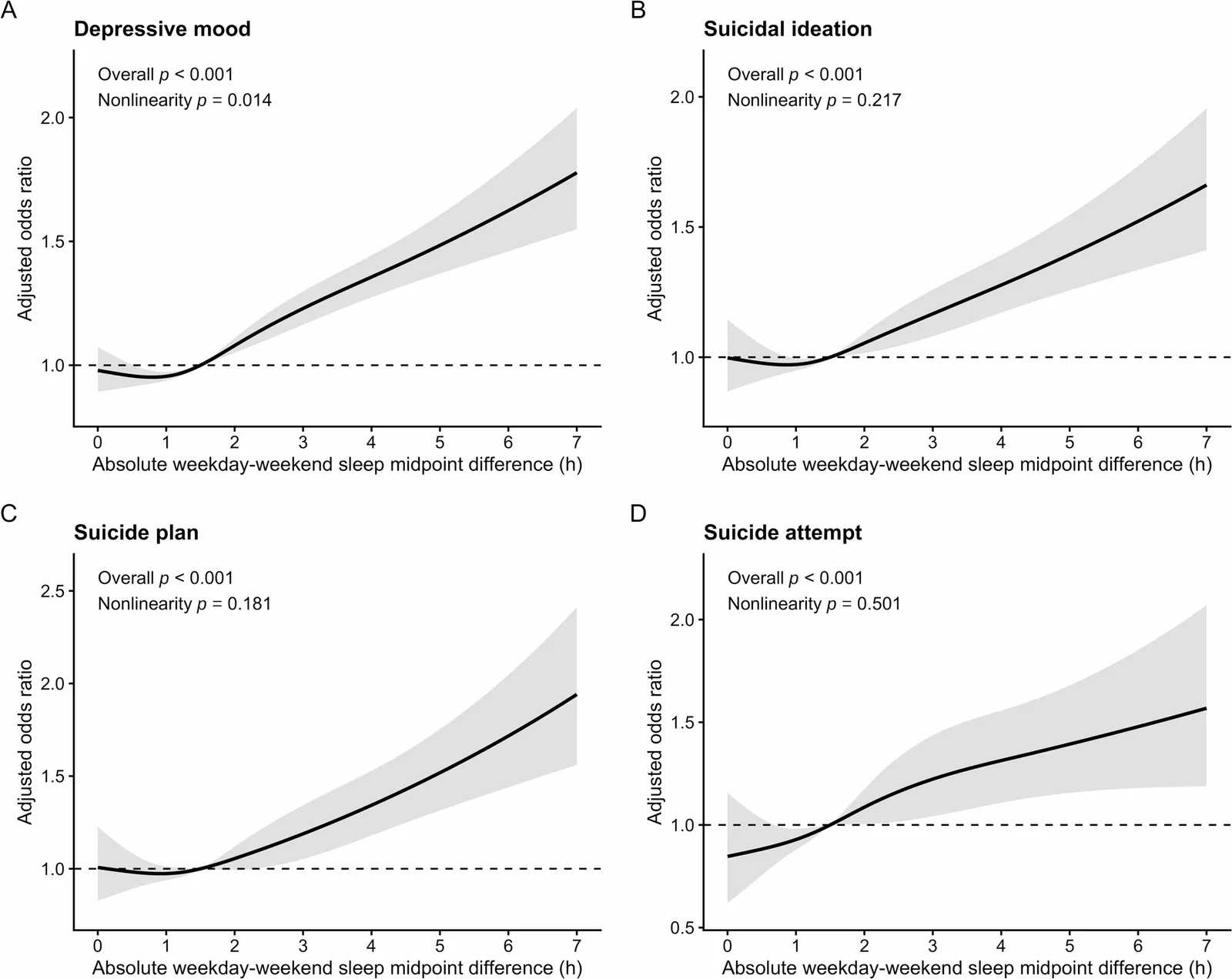
Adjusted associations of the absolute weekday–weekend sleep midpoint difference with depressive mood and suicidal outcomes. Survey-weighted restricted cubic spline curves show the associations of the continuous absolute weekday–weekend sleep midpoint difference with (**A**) depressive mood, (**B**) suicidal ideation, (**C**) suicide plan, and (**D**) suicide attempt. Solid lines represent adjusted odds ratios, shaded areas represent 95% confidence intervals, and horizontal dashed lines indicate an odds ratio of 1. Four knots were placed at the survey-weighted 5th, 35th, 65th, and 95th percentiles of the midpoint difference, and the survey-weighted median was used as the reference value. Models were adjusted for sex, school grade, categorical socioeconomic status, categorical academic performance, MSFsc, and weekly average sleep duration; the latter two sleep descriptors were entered as linear terms. Curves span the survey-weighted 1st to 99th percentiles, while all eligible observations remained included in model fitting. The analytic sample included 48,416 participants

In the primary restricted cubic spline analyses, MSFsc showed significant overall associations with depressive mood, suicidal ideation, and suicide plan (all *p* < 0.001), whereas the overall association with suicide attempt did not reach statistical significance (*p* = 0.054; Fig. 2). Average weekly sleep duration and the absolute weekday–weekend sleep midpoint difference showed significant overall associations with all four mental health outcomes (all *p* < 0.001; Table 2; Figs. 3 and 4).

**Table 2:** Overall and nonlinear associations of continuous sleep descriptors with mental health outcomes

Exposure	Outcome	*N*	Overall F (df1, df2)	Overall *p* value	Nonlinearity F (df1, df2)	Nonlinearity *p* value
MSFsc	Depressive mood	48,416	15.35 (3, 675)	< 0.001	3.96 (2, 675)	0.019
MSFsc	Suicidal ideation	48,416	5.67 (3, 675)	< 0.001	5.40 (2, 675)	0.005
MSFsc	Suicide plan	48,416	11.59 (3, 675)	< 0.001	11.29 (2, 675)	< 0.001
MSFsc	Suicide attempt	48,416	2.55 (3, 675)	0.054	0.01 (2, 675)	0.993
Weekly average sleep duration	Depressive mood	48,416	178.86 (3, 675)	< 0.001	19.77 (2, 675)	< 0.001
Weekly average sleep duration	Suicidal ideation	48,416	175.56 (3, 675)	< 0.001	26.60 (2, 675)	< 0.001
Weekly average sleep duration	Suicide plan	48,416	90.78 (3, 675)	< 0.001	27.52 (2, 675)	< 0.001
Weekly average sleep duration	Suicide attempt	48,416	73.14 (3, 675)	< 0.001	24.14 (2, 675)	< 0.001
Absolute weekday–weekend sleep midpoint difference	Depressive mood	48,416	36.15 (3, 675)	< 0.001	4.30 (2, 675)	0.014
Absolute weekday–weekend sleep midpoint difference	Suicidal ideation	48,416	14.72 (3, 675)	< 0.001	1.53 (2, 675)	0.217
Absolute weekday–weekend sleep midpoint difference	Suicide plan	48,416	12.65 (3, 675)	< 0.001	1.72 (2, 675)	0.181
Absolute weekday–weekend sleep midpoint difference	Suicide attempt	48,416	6.57 (3, 675)	< 0.001	0.69 (2, 675)	0.501

Values are from survey-weighted logistic regression models using restricted cubic splines. Each model included the focal sleep descriptor as a spline and adjusted for sex, school grade, five-level categorical socioeconomic status, five-level categorical academic performance, and the other two sleep descriptors as linear terms. Four knots were placed at the survey-weighted 5th, 35th, 65th, and 95th percentiles, and the survey-weighted median was used as the reference value. Overall *p* values are from joint design-based Wald tests of all three spline terms; nonlinearity *p* values are from joint tests of the two nonlinear spline terms. All models used the final complete-case sample (*N* = 48,416). MSFsc, corrected midpoint of sleep on free days

Significant nonlinearity was observed for the associations of MSFsc with depressive mood (*p* = 0.019), suicidal ideation (*p* = 0.005), and suicide plan (*p* < 0.001), but not with suicide attempt (*p* = 0.993). Average weekly sleep duration showed significant nonlinear associations with all four outcomes (all *p* < 0.001). For the absolute weekday–weekend sleep midpoint difference, significant nonlinearity was observed for depressive mood (*p* = 0.014), but not for suicidal ideation (*p* = 0.217), suicide plan (*p* = 0.181), or suicide attempt (*p* = 0.501).

Across the adjusted curves, shorter average weekly sleep duration was consistently associated with higher odds of all four outcomes. Larger absolute weekday–weekend sleep midpoint differences were also generally associated with higher odds, whereas the patterns for MSFsc were less uniform and differed across outcomes.

### Categorical MSFsc-based sleep-timing groups

In the secondary categorical analyses, MSFsc-based sleep-timing group was associated with each mental health outcome in the fully adjusted models (all omnibus *p* < 0.001; Table 3). Compared with the morning group, the intermediate group had lower adjusted odds of depressive mood (aOR, 0.85; 95% CI, 0.80–0.91), suicidal ideation (aOR, 0.84; 95% CI, 0.77–0.92), suicide plan (aOR, 0.66; 95% CI, 0.59–0.75), and suicide attempt (aOR, 0.73; 95% CI, 0.62–0.86). The evening group also had lower adjusted odds of depressive mood (aOR, 0.88; 95% CI, 0.81–0.96), suicide plan (aOR, 0.75; 95% CI, 0.63–0.89), and suicide attempt (aOR, 0.71; 95% CI, 0.57–0.88) than the morning group. For suicidal ideation, the evening-versus-morning estimate was closer to the null and its confidence interval included 1.00 (aOR, 0.94; 95% CI, 0.83–1.06). Across these adjusted models, female sex and the lowest socioeconomic-status and academic-performance categories were generally associated with higher odds of adverse mental health outcomes, whereas higher school grade was associated with lower odds.

**Table 3:** Fully adjusted associations of MSFsc-based sleep-timing groups with depressive mood and suicidal outcomes

MSFsc-based sleep-timing group	Depressive mood aOR (95% CI); *p*	Suicidal ideation aOR (95% CI); *p*	Suicide plan aOR (95% CI); *p*	Suicide attempt aOR (95% CI); *p*
Morning (reference)	1.00 (reference)	1.00 (reference)	1.00 (reference)	1.00 (reference)
Intermediate vs. Morning	0.85 (0.80–0.91); <0.001	0.84 (0.77–0.92); <0.001	0.66 (0.59–0.75); <0.001	0.73 (0.62–0.86); <0.001
Evening vs. Morning	0.88 (0.81–0.96); 0.005	0.94 (0.83–1.06); 0.310	0.75 (0.63–0.89); 0.001	0.71 (0.57–0.88); 0.002
Omnibus *p*	< 0.001	< 0.001	< 0.001	< 0.001

Adjusted odds ratios were estimated using survey-weighted logistic regression in the final complete-case sample (*N* = 48,416). Models adjusted for sex, school grade, five-level categorical socioeconomic status, five-level categorical academic performance, weekly average sleep duration, and the absolute weekday–weekend sleep midpoint difference. The morning group was the reference category. The omnibus *p* value jointly tests the two nonreference sleep-timing group coefficients. Estimates are mutually adjusted conditional associations and should not be interpreted as independent causal effects or mediation estimates. aOR, adjusted odds ratio; CI, confidence interval; MSFsc, corrected midpoint of sleep on free days

The sequential models showed that the sleep-timing group estimates varied according to the adjustment set (Supplementary Table S4). In Model A, which adjusted for sociodemographic covariates, the evening group had higher odds than the morning group of depressive mood (OR, 1.27; 95% CI, 1.19–1.35), suicidal ideation (OR, 1.38; 95% CI, 1.25–1.52), and suicide plan (OR, 1.20; 95% CI, 1.04–1.38), whereas the confidence interval for suicide attempt included 1.00 (OR, 1.19; 95% CI, 0.98–1.44). Adding average weekly sleep duration in Model B shifted the evening-group estimates toward the null or below 1.00. Adding the absolute midpoint difference alone in Model C produced outcome-dependent changes, with all four evening-group estimates remaining above 1.00. When both sleep descriptors were included in Model D, all intermediate- and evening-group estimates were below 1.00, except that the confidence interval for the evening-versus-morning contrast for suicidal ideation included the null. Changes across these models describe conditional associations under different adjustment sets and should not be interpreted as evidence of mediation.

### Combined sleep-timing group–sleep duration profiles

In the exploratory joint-profile analyses, the morning group with 8 to < 9 h of sleep was used as the reference profile. Sleep duration of < 5 h was associated with higher adjusted odds of all four mental health outcomes across all three MSFsc-based sleep-timing groups (Supplementary Table S9). In the morning group, the adjusted odds ratios for < 5 h of sleep were 3.13 (95% CI, 2.46–3.98) for depressive mood, 3.39 (95% CI, 2.52–4.57) for suicidal ideation, 4.17 (95% CI, 2.81–6.17) for suicide plan, and 4.50 (95% CI, 2.65–7.62) for suicide attempt. The corresponding estimates in the intermediate group were 2.03 (95% CI, 1.78–2.32), 2.95 (95% CI, 2.43–3.58), 2.39 (95% CI, 1.77–3.23), and 3.09 (95% CI, 2.12–4.50), respectively. In the evening group, the corresponding estimates were 2.42 (95% CI, 2.07–2.82), 3.44 (95% CI, 2.77–4.26), 3.22 (95% CI, 2.35–4.40), and 3.02 (95% CI, 1.96–4.64), respectively. Associations were generally smaller at longer sleep durations, although the pattern was not strictly monotonic across all sleep-timing groups and outcomes.

Among the 72 direct contrasts between sleep-timing groups within sleep-duration strata, four remained statistically significant after Benjamini–Hochberg false-discovery-rate adjustment (Supplementary Table S8). The intermediate group had lower odds of depressive mood than the morning group among participants sleeping < 5 h (OR, 0.65; 95% CI, 0.52–0.82; *q* = 0.006) and 8 to < 9 h (OR, 0.75; 95% CI, 0.65–0.87; *q* = 0.004). Among participants sleeping 7 to < 8 h, the evening group had higher odds of suicidal ideation than the intermediate group (OR, 1.40; 95% CI, 1.13–1.74; *q* = 0.044), whereas the intermediate group had lower odds of suicide plan than the morning group (OR, 0.52; 95% CI, 0.39–0.71; *q* = 0.002). No direct contrast for suicide attempt remained statistically significant after false-discovery-rate adjustment. The omnibus sleep-timing group–sleep duration interaction was statistically significant for depressive mood (*p* = 0.006), but not for suicidal ideation (*p* = 0.089), suicide plan (*p* = 0.426), or suicide attempt (*p* = 0.754). These results provide limited and outcome-specific evidence of effect modification and should be interpreted as exploratory.

### Sensitivity analyses

The categorical findings were unchanged when MSFsc-based sleep-timing groups were redefined using survey-weighted 20th and 80th percentile cutoffs or survey-weighted 25th and 75th percentile cutoffs. Neither alternative classification changed the statistical significance of any of the eight sleep-timing group contrasts or the four omnibus tests, and the largest corresponding changes in odds ratios were modest (Supplementary Table S5). In the common sample of 47,565 participants, additional adjustment for weekly academic-purpose sitting time did not change the statistical significance of any overall-association or nonlinearity test across the 12 spline models. The maximum ratio change in the corresponding predicted odds-ratio curves was 1.021 (Supplementary Table S5).

Excluding the 514 observations with extreme extended-scale MSFsc values below 24 or above 36 h left 47,902 participants. The overall findings for average weekly sleep duration and the absolute weekday–weekend sleep midpoint difference were largely preserved. The associations of MSFsc with depressive mood, suicidal ideation, and suicide plan also remained statistically significant. In contrast, the MSFsc association with suicide attempt changed from a nonsignificant overall association in the primary analysis (*p* = 0.054) to a significant overall and nonlinear association after exclusion of the extreme values (overall *p* < 0.001; nonlinearity *p* = 0.007), indicating that this specific result was sensitive to the extreme MSFsc observations (Supplementary Table S5).

Among the 48,426 participants with valid sleep-derived variables, 4,467 had weekend bedtime responses between 04:00 and 19:59; 4,465 of these participants were included in the final complete-case sample. These flagged responses were present in 403 of 431 observations with extended-scale MSFsc values below 24 h and in all 83 observations with MSFsc values above 36 h. Results were also broadly consistent when the absolute midpoint difference was replaced with the signed weekend-minus-weekday midpoint difference, when 4,465 participants with weekend bedtime responses between 04:00 and 19:59 were excluded (*N* = 43,951), and when weekday and weekend sleep durations were restricted to 3–14 h (*N* = 46,993). In these analyses, later weekend relative to weekday sleep midpoint was associated with higher odds of all four outcomes, and the intermediate group generally retained lower odds than the morning group. The evening-versus-morning estimates were more variable and were close to the null for some outcomes, particularly after the plausible-duration restriction (Supplementary Table S6).

In models examining weekday and weekend sleep duration separately, longer sleep duration on both weekdays and weekends was associated with lower odds of all four mental health outcomes. The inverse estimates were consistently larger in magnitude for weekday sleep duration than for weekend sleep duration. Models that included weekday sleep duration and weekend sleep extension together showed inverse associations of both measures with the outcomes, although these mutually adjusted estimates remain conditional because the sleep measures share component timing variables (Supplementary Table S7).

## Discussion

In this nationally representative sample of Korean adolescents, we began with the question of whether the higher mental health risk observed among adolescents with later sleep timing reflected sleep timing itself or the shorter sleep duration and larger weekday–weekend sleep midpoint differences that commonly accompanied it. The overall pattern was more consistent with the latter interpretation. Before conditioning on the other sleep descriptors, the evening MSFsc-based sleep-timing group had higher odds than the morning group of depressive mood, suicidal ideation, and suicide plan, whereas the confidence interval for suicide attempt included 1.00. In the primary continuous analyses, shorter average weekly sleep duration and larger absolute weekday–weekend sleep midpoint differences showed overall associations with all four mental health outcomes, whereas the associations involving MSFsc were less uniform and were less robust for suicide attempt. Sleep duration showed nonlinear associations with all four outcomes, with markedly higher odds at shorter durations, whereas significant nonlinearity of the midpoint difference was observed only for depressive mood. Taken together, these findings shift the main interpretive emphasis toward insufficient sleep and weekday–weekend sleep-timing discrepancy, while retaining later sleep timing as an important component of the overall sleep pattern.

The categorical sleep-timing findings require interpretation at two levels. In Model A, before conditioning on the other sleep descriptors, the evening MSFsc-based sleep-timing group showed higher odds of depressive mood, suicidal ideation, and suicide plan than the morning group, a pattern broadly consistent with previous meta-analytic evidence linking eveningness with depressive symptoms [24]. In contrast, Model D simultaneously conditioned on average weekly sleep duration and the absolute weekday–weekend sleep midpoint difference. These variables were derived from overlapping weekday and weekend sleep-timing information, and sleep duration was incorporated directly into the MSFsc correction formula. Although the correlations among the three sleep descriptors were modest and the variance inflation factors ranged from 1.250 to 1.594, these diagnostics address conventional variance inflation and do not remove the structural coupling created by shared component variables. Conditioning on these related and potentially downstream sleep descriptors may therefore have contributed to suppression and to the reversal of several evening-versus-morning estimates from above to below 1.00. Accordingly, the fully adjusted estimates should not be interpreted as evidence that later sleep timing is protective or that adolescents in the morning group are inherently more vulnerable. They are more appropriately understood as conditional associations under a particular adjustment set rather than as direct causal effects or evidence of mediation.

Average weekly sleep duration showed one of the clearest and most consistent patterns in this study. It was associated with all four mental health outcomes in the primary continuous analyses, and significant nonlinearity was observed for each outcome, with the adjusted odds increasing markedly at shorter sleep durations. This pattern is consistent with previous dose–response evidence linking short sleep duration with adolescent suicidal ideation and suicidal behavior [23]. Similar associations between insufficient sleep and suicidality have been reported among Korean adolescents [37, 38]. The sensitivity analyses further showed that longer sleep duration on both weekdays and weekends was associated with lower odds of all four outcomes, although the inverse estimates were numerically larger for weekday sleep duration. This finding is broadly consistent with a previous study of university students in which shorter weekday sleep was associated with poorer self-rated health, whereas chronotype and weekday–weekend differences were not associated after weekday sleep was considered; however, direct comparison is limited by differences in age and outcome [39]. Experimental and review evidence suggests that sleep restriction may worsen mood, impair emotion regulation, and increase suicide risk, providing plausible pathways linking insufficient sleep with depressive mood and suicidal outcomes [40, 41].

The absolute weekday–weekend sleep midpoint difference also showed a consistent overall association with all four mental health outcomes. However, its functional form differed from that of sleep duration: significant nonlinearity was observed only for depressive mood, whereas the associations with suicidal ideation, suicide plan, and suicide attempt were generally monotonic over the observed range. These findings are broadly consistent with previous evidence linking weekday–weekend differences in sleep timing with poorer mental health among young people, including depressive symptoms [26–28]. Nevertheless, the midpoint difference used in this study was calculated from self-reported weekday and weekend sleep midpoints and should not be interpreted as a direct measure of internal circadian phase displacement [30, 31]. The mutually adjusted estimates also remain conditional because the midpoint difference, MSFsc, and sleep duration share component sleep-timing information. Thus, the present findings support the relevance of weekday–weekend sleep-timing discrepancy as a behavioral marker but do not establish an independent causal effect of circadian misalignment.

The combined sleep-timing group–sleep duration analysis was exploratory and was intended to describe readily interpretable sleep profiles rather than to establish effect modification. Very short sleep duration was associated with elevated odds of all four mental health outcomes across all three MSFsc-based sleep-timing groups. However, the formal interaction was statistically significant only for depressive mood, and only four of the 72 direct within-stratum contrasts remained significant after false-discovery-rate adjustment; no direct contrast for suicide attempt remained significant. These findings do not support a general conclusion that one sleep-timing group is consistently more vulnerable to short sleep than another. The relatively large estimates observed for the morning group with less than 5 h of sleep also require caution because the MSFsc correction formula incorporates sleep duration. In particular, very short weekday sleep may shift corrected MSFsc earlier and thereby influence both sleep-timing group assignment and the resulting joint-profile estimates. The combined-profile analysis therefore complements the primary continuous analyses by illustrating patterns across sleep-timing and sleep-duration combinations, but it does not provide evidence that susceptibility to short sleep differs consistently across MSFsc-based sleep-timing groups.

The Korean educational environment provides an important structural context for these findings. Adolescents undergo a developmental shift toward later sleep timing, while school attendance continues to require relatively early weekday wake times. In Korea, regional studies have documented early school start times before delayed-start initiatives, and subsequent policies delaying school start times have been associated with longer sleep opportunities among adolescents [16, 20, 21]. As a general illustration of Korean secondary-school routines, the school day may involve arrival around 08:00–09:00, followed by regular classes until the mid- to late afternoon, approximately 15:00–17:00. Exact schedules vary by school level, region, school, grade, and day of the week. School-based after-school programs or supervised self-study may extend students’ time at school beyond regular class hours. In addition, a substantial proportion of Korean middle- and high-school students participate in private education outside regular school hours, which may further constrain evening time and sleep opportunity [22]. These structural demands could contribute to both short weekday sleep and larger weekday–weekend sleep-timing differences. Academic and achievement-related stress may also be associated with mental health independently of sleep; however, the present study could not separate these direct associations from pathways operating through sleep. Because the KYRBS did not provide individual school start and end times or private-academy attendance duration, these mechanisms could not be evaluated directly. Additional adjustment for academic-purpose sitting time produced minimal changes in the spline results, but this variable was only a broad proxy and does not exclude residual confounding by school schedules or academic demands.

This study has several strengths. First, it used a large, nationally representative sample of Korean middle- and high-school students and accounted for the complex sampling design of the KYRBS. Second, the primary analyses retained MSFsc, average weekly sleep duration, and the absolute weekday–weekend sleep midpoint difference as continuous variables and used restricted cubic splines to characterize potentially nonlinear associations rather than relying only on arbitrary categories. Third, the study examined four related but clinically distinct mental health outcomes, ranging from depressive mood to suicidal ideation, suicide plan, and suicide attempt. Fourth, correlations, variance inflation factors, and sequential adjustment models were used to make the shared derivation and conditional interpretation of the sleep descriptors explicit. Fifth, multiple sensitivity analyses evaluated alternative MSFsc cutoffs, extreme MSFsc values, potentially implausible weekend bedtimes, plausible sleep-duration ranges, signed midpoint differences, weekday and weekend sleep duration, and additional adjustment for academic-purpose sitting time. Finally, the exploratory combined-profile analyses were supplemented by direct within-stratum contrasts and false-discovery-rate adjustment, allowing the joint-profile findings to be interpreted more cautiously.

However, this study also has several limitations. First, the cross-sectional design precludes determination of temporal ordering and causal inference. Depressive mood or suicidal outcomes may contribute to sleep disturbances, and the present analyses cannot establish whether sleep duration or the weekday–weekend sleep midpoint difference lies on a causal pathway between sleep timing and mental health. The sequential models therefore describe changes in conditional associations rather than formal mediation. Potential mediation should be evaluated in future longitudinal studies with temporally ordered measurements. Second, sleep timing and mental health outcomes were self-reported and may be affected by recall or reporting error. Previous adolescent studies have documented discrepancies between self-reported and actigraphy-based sleep duration [42]. Third, MSFsc was reconstructed from the weekday and weekend sleep-timing items available in the KYRBS rather than measured using the full Munich ChronoType Questionnaire or a morningness–eveningness preference instrument. It may therefore reflect state-dependent sleep timing and external constraints, including school schedules and academic demands, as well as endogenous circadian preference. The percentile-based sleep-timing groups were also defined using sample-specific cutoffs and should not be interpreted as validated clinical chronotype categories; categorization may separate participants with very similar MSFsc values. Fourth, MSFsc, sleep duration, and the weekday–weekend sleep midpoint difference were mathematically dependent because they were derived from overlapping sleep-timing variables. Although conventional collinearity diagnostics showed limited variance inflation, this structural coupling complicates interpretation of mutually adjusted estimates and may contribute to suppression or coefficient reversal. Fifth, participants excluded because of incomplete or invalid sleep-derived data had higher prevalence of depressive mood and suicidal outcomes than included participants, raising the possibility of selection bias. The selective exclusion of these higher-risk adolescents may have led to underestimation of outcome prevalences and attenuation of the observed sleep–mental health associations. In addition, because the KYRBS is a school-based survey, the findings may not generalize to adolescents who were not attending school. Finally, individual school schedules, private-academy attendance duration, and academic stress were not directly measured. Academic-purpose sitting time was only a broad proxy, and residual confounding by educational and behavioral factors cannot be excluded.

## Conclusions

In this nationally representative sample of Korean adolescents, shorter sleep duration and larger absolute weekday–weekend sleep midpoint differences showed the most consistent associations with depressive mood and suicidal outcomes. Later MSFsc-based sleep timing was also associated with higher odds of several outcomes before conditioning on the other sleep descriptors, but these estimates changed substantially after adjustment for sleep duration and the midpoint difference. Because the sleep descriptors were derived from overlapping sleep-timing information, the fully adjusted estimates should be interpreted as conditional associations rather than as independent causal effects. Very short sleep duration was associated with elevated odds across all sleep-timing groups, although evidence that these associations differed consistently by sleep-timing group was limited. These findings highlight adequate sleep duration and smaller weekday–weekend sleep-timing discrepancies as potentially relevant targets for future adolescent mental health research and prevention efforts. Longitudinal studies using independently measured circadian and sleep characteristics are needed to clarify temporal and causal pathways.

## Supplementary Information

Below is the link to the electronic supplementary material.

Supplementary Material 1

## Data Availability

The data analyzed in this study are publicly available from the Korea Disease Control and Prevention Agency through the Korea Youth Risk Behavior Survey website (https://www.kdca.go.kr/yhs/home.jsp). Researchers can apply for access according to the standard data use procedures of the Korea Disease Control and Prevention Agency.
