# Supplementary material for: Associations of sleep duration and sleep-timing patterns with depressive mood and suicidal outcomes in Korean adolescents
Source: BMC Psychiatry. 2026 Oct 13;26:893. doi: 10.1186/s12888-026-08706-6 (PMC13757211; doi:10.1186/s12888-026-08706-6)
Supplement: Supplementary file 1 [file 12888_2026_8706_MOESM1_ESM.docx]

**Supplementary Table S1. Survey-weighted comparison of participants included in and excluded from the valid sleep-derived sample**

| **Characteristic** | **Category** | **Included (*n* = 48,426)** | **Excluded (*n* = 5,744)** | ***p* value** | **Absolute standardized difference** |
| --- | --- | --- | --- | --- | --- |
| Age, years |  | 14.99 (0.02) | 14.80 (0.03) | <0.001 | 0.106 |
| Sex | Male | 51.0 (1.1) | 54.6 (1.2) | <0.001 | 0.073 |
|  | Female | 49.0 (1.1) | 45.4 (1.2) |  | 0.073 |
| School level | High school | 49.2 (0.8) | 44.7 (1.1) | <0.001 | 0.090 |
|  | Middle school | 50.8 (0.8) | 55.3 (1.1) |  | 0.090 |
| Grade | Middle school, grade 1 | 17.2 (0.3) | 20.1 (0.6) | <0.001 | 0.074 |
|  | Middle school, grade 2 | 16.8 (0.3) | 18.1 (0.6) |  | 0.033 |
|  | Middle school, grade 3 | 16.8 (0.3) | 17.1 (0.6) |  | 0.009 |
|  | High school, grade 1 | 16.2 (0.3) | 14.0 (0.5) |  | 0.062 |
|  | High school, grade 2 | 16.1 (0.3) | 16.2 (0.6) |  | 0.000 |
|  | High school, grade 3 | 16.9 (0.3) | 14.6 (0.6) |  | 0.062 |
| Socioeconomic status | Level 1 (High) | 11.0 (0.2) | 14.0 (0.5) | <0.001 | 0.091 |
|  | Level 2 | 32.4 (0.3) | 29.0 (0.6) |  | 0.072 |
|  | Level 3 | 46.3 (0.3) | 43.8 (0.7) |  | 0.051 |
|  | Level 4 | 8.8 (0.2) | 9.9 (0.4) |  | 0.037 |
|  | Level 5 (Low) | 1.6 (0.1) | 3.3 (0.3) |  | 0.114 |
| Academic performance | Level 1 (High) | 12.2 (0.2) | 12.5 (0.5) | <0.001 | 0.011 |
|  | Level 2 | 26.3 (0.2) | 22.3 (0.5) |  | 0.093 |
|  | Level 3 | 29.0 (0.2) | 28.2 (0.6) |  | 0.018 |
|  | Level 4 | 23.3 (0.2) | 23.6 (0.6) |  | 0.007 |
|  | Level 5 (Low) | 9.2 (0.1) | 13.4 (0.5) |  | 0.131 |
| Depressive mood | Yes | 25.2 (0.2) | 29.8 (0.6) | <0.001 | 0.102 |
| Suicidal ideation | Yes | 11.2 (0.2) | 15.5 (0.5) | <0.001 | 0.128 |
| Suicide plan | Yes | 4.1 (0.1) | 7.8 (0.4) | <0.001 | 0.158 |
| Suicide attempt | Yes | 2.3 (0.1) | 6.1 (0.4) | <0.001 | 0.189 |

Values are survey-weighted means (standard errors) for age and survey-weighted percentages (standard errors) for categorical variables. The observed age range was 12–18 years. *p* values were obtained from a design-adjusted Wald test for age and Rao–Scott design-adjusted tests for categorical variables. Absolute standardized differences were calculated using the pooled survey-weighted variance for age and pooled binary proportions for each categorical level. Values below 0.10 are commonly interpreted as indicating small between-group differences. Mental-health rows show the affirmative category. Variable-specific missing values, if any, were excluded from the corresponding estimate.

**Supplementary Table S2. Distribution of corrected mid-sleep on free days and MSFsc-based sleep-timing group classification thresholds**

**Panel A. Distributional summary of MSFsc**

| **Statistic** | **Extended-hour value** | **Clock-time equivalent (24-h clock)** |
| --- | --- | --- |
| Analytic sample, *N* | 48,426 |  |
| Unweighted mean (SD) | 28.8289 (1.6776) | 04:50 |
| Survey-weighted mean (SE) | 28.8421 (0.0096) | 04:51 |
| Minimum | 13.1667 | 13:10 |
| 5th percentile | 26.6667 | 02:40 |
| 20th percentile cutoff used | 27.7619 | 03:46 |
| 25th percentile | 27.9762 | 03:59 |
| Median | 28.8452 | 04:51 |
| 75th percentile | 29.7262 | 05:44 |
| 80th percentile cutoff used | 29.9524 | 05:57 |
| 95th percentile | 31.1667 | 07:10 |
| Maximum | 40.7262 | 16:44 (+1 day) |

**Panel B. MSFsc-based sleep-timing group classification**

| **Sleep-timing group** | **Classification rule** | **Clock-time interpretation** | ***n*** | **Percent** |
| --- | --- | --- | --- | --- |
| Morning group | MSFsc < P20 | MSFsc < 03:46 | 9,643 | 19.9 |
| Intermediate group | P20 ≤ MSFsc ≤ P80 | 03:46 ≤ MSFsc ≤ 05:57 | 29,123 | 60.1 |
| Evening group | MSFsc > P80 | MSFsc > 05:57 | 9,660 | 19.9 |

MSFsc denotes corrected mid-sleep on free days. Distributional summaries and sleep-timing group counts are based on participants with valid sleep-derived variables (*N* = 48,426). The exact unweighted 20th and 80th percentile values were 27.761905 and 29.952381 extended hours, respectively; these are displayed to four decimal places in Panel A and correspond to approximately 03:46 and 05:57 on the 24-hour clock. The verified boundary convention reproducing the stored sleep-timing group classification was MSFsc < P20 for the morning group, P20 ≤ MSFsc ≤ P80 for the intermediate group, and MSFsc > P80 for the evening group. The original SPSS syntax stored the lower cutoff to four decimal places and used MSFsc <= 27.7619; this produced the same stored groups in the present dataset. Percentages may not sum to 100 because of rounding. The full observed range is shown; sensitivity analyses separately address potentially implausible weekend sleep-timing responses.

**Supplementary Table S3.** **Survey-weighted correlations and collinearity diagnostics among sleep descriptors**

**Panel A. Correlation matrix**

| **Variable** | **1** | **2** | **3** |
| --- | --- | --- | --- |
| MSFsc | 1.000 | -0.288 (<0.001) | 0.486 (<0.001) |
| Weekly average sleep duration | -0.288 (<0.001) | 1.000 | 0.160 (<0.001) |
| Absolute weekday–weekend midpoint difference | 0.486 (<0.001) | 0.160 (<0.001) | 1.000 |

**Panel B. Variance inflation factors**

| **Variable** | **Auxiliary-model R-squared** | **Tolerance** | **VIF** |
| --- | --- | --- | --- |
| MSFsc | 0.373 | 0.627 | 1.594 |
| Weekly average sleep duration | 0.200 | 0.800 | 1.250 |
| Absolute weekday–weekend midpoint difference | 0.334 | 0.666 | 1.501 |

Panel A reports survey-weighted Pearson correlation coefficients; design-adjusted *p* values are shown in parentheses. Variables are numbered as follows: 1, corrected mid-sleep on free days (MSFsc); 2, weekly average sleep duration; and 3, absolute weekday–weekend sleep midpoint difference. Panel B reports tolerance and variance inflation factors (VIFs) calculated from sampling-weighted auxiliary linear regressions in which each sleep descriptor was regressed on the other two. Tolerance was calculated as 1 − R², and VIF as the reciprocal of tolerance using unrounded values; displayed values were rounded to three decimal places. Analyses used the final complete-case model sample (*N* = 48,416). Descriptive analyses of the MSFsc distribution used all participants with valid sleep-derived variables (*N* = 48,426).

**Supplementary Table S4. Sequentially adjusted associations of MSFsc-based sleep-timing group with depressive mood and suicidal outcomes**

**Panel A. Sleep-timing group contrasts**

| **Outcome** | **Contrast** | **Model A** | **Model B** | **Model C** | **Model D** |
| --- | --- | --- | --- | --- | --- |
| Depressive mood | Evening vs Morning | 1.269 (1.192–1.351) | 1.080 (1.011–1.154) | 1.277 (1.180–1.382) | 0.883 (0.809–0.964) |
|  | Intermediate vs Morning | 1.018 (0.965–1.075) | 0.911 (0.861–0.964) | 1.020 (0.964–1.079) | 0.854 (0.805–0.907) |
| Suicidal ideation | Evening vs Morning | 1.377 (1.247–1.521) | 1.102 (0.995–1.219) | 1.483 (1.318–1.669) | 0.938 (0.829–1.062) |
|  | Intermediate vs Morning | 1.025 (0.945–1.112) | 0.882 (0.811–0.958) | 1.047 (0.963–1.138) | 0.839 (0.769–0.915) |
| Suicide plan | Evening vs Morning | 1.195 (1.038–1.376) | 0.938 (0.812–1.083) | 1.189 (0.992–1.426) | 0.746 (0.625–0.889) |
|  | Intermediate vs Morning | 0.835 (0.738–0.944) | 0.710 (0.627–0.804) | 0.833 (0.734–0.946) | 0.664 (0.585–0.753) |
| Suicide attempt | Evening vs Morning | 1.191 (0.982–1.444) | 0.917 (0.750–1.122) | 1.148 (0.918–1.436) | 0.710 (0.570–0.885) |
|  | Intermediate vs Morning | 0.934 (0.794–1.097) | 0.787 (0.669–0.926) | 0.924 (0.785–1.088) | 0.733 (0.623–0.862) |

**Panel B. Omnibus *p* values for Sleep-timing group**

| **Outcome** | **Model A** | **Model B** | **Model C** | **Model D** |
| --- | --- | --- | --- | --- |
| Depressive mood | <0.001 | <0.001 | <0.001 | <0.001 |
| Suicidal ideation | <0.001 | <0.001 | <0.001 | <0.001 |
| Suicide plan | <0.001 | <0.001 | <0.001 | <0.001 |
| Suicide attempt | 0.006 | 0.005 | 0.039 | <0.001 |

Values are odds ratios (95% confidence intervals) from survey-weighted logistic regression models, with the morning group as the reference category. Model A adjusted for sex, grade, categorical perceived socioeconomic status, and categorical academic performance. Model B additionally included weekly average sleep duration. Model C additionally included the absolute weekday–weekend sleep midpoint difference. Model D included both weekly average sleep duration and the absolute midpoint difference. The omnibus *p* values test the two sleep-timing group coefficients jointly. All models used the same final complete-case sample (*N* = 48,416). Changes in estimates across models describe conditional associations under different adjustment sets and should not be interpreted as evidence of mediation.

**Supplementary Table S5. Summary of R-based sensitivity analyses for sleep-timing classification and nonlinear models**

**Panel A. Alternative sleep-timing group classification thresholds**

| **Sensitivity definition** | **Cutoffs, extended hour (clock time)** | **Model *N*** | **Term significance changes, *n*/8** | **Omnibus significance changes, *n*/4** | **Largest OR-ratio change** |
| --- | --- | --- | --- | --- | --- |
| Survey-weighted P20/P80 | 27.7857 (03:47) / 29.9643 (05:58) | 48,416 | 0/8 | 0/4 | 1.019 |
| Survey-weighted P25/P75 | 28.0000 (04:00) / 29.7381 (05:44) | 48,416 | 0/8 | 0/4 | 1.107 |

Panel A compares the primary unweighted P20/P80 sleep-timing group classification with survey-weighted P20/P80 and P25/P75 classifications. Term-level comparisons comprise Evening versus Morning and Intermediate versus Morning for four outcomes. The largest OR-ratio change is the largest symmetric ratio between a sensitivity estimate and its corresponding primary estimate.

**Panel B. Exclusion of extreme MSFsc values**

| **Exposure** | **Outcome** | **Primary *N*** | **Restricted *N*** | **Primary overall *p*** | **Restricted overall *p*** | **Primary nonlinearity *p*** | **Restricted nonlinearity *p*** | **Maximum OR-ratio change** | **Overall significance changed** | **Nonlinearity significance changed** |
| --- | --- | --- | --- | --- | --- | --- | --- | --- | --- | --- |
| MSFsc | Depressive mood | 48,416 | 47,902 | <0.001 | <0.001 | 0.019 | 0.036 | 1.208 | No | No |
| MSFsc | Suicidal ideation | 48,416 | 47,902 | <0.001 | <0.001 | 0.005 | <0.001 | 1.432 | No | No |
| MSFsc | Suicide plan | 48,416 | 47,902 | <0.001 | <0.001 | <0.001 | <0.001 | 1.829 | No | No |
| MSFsc | Suicide attempt | 48,416 | 47,902 | 0.054 | <0.001 | 0.993 | 0.007 | 2.985 | Yes | Yes |
| Weekly average sleep duration | Depressive mood | 48,416 | 47,902 | <0.001 | <0.001 | <0.001 | <0.001 | 1.121 | No | No |
| Weekly average sleep duration | Suicidal ideation | 48,416 | 47,902 | <0.001 | <0.001 | <0.001 | <0.001 | 1.052 | No | No |
| Weekly average sleep duration | Suicide plan | 48,416 | 47,902 | <0.001 | <0.001 | <0.001 | <0.001 | 1.072 | No | No |
| Weekly average sleep duration | Suicide attempt | 48,416 | 47,902 | <0.001 | <0.001 | <0.001 | <0.001 | 1.285 | No | No |
| Absolute weekday–weekend midpoint difference | Depressive mood | 48,416 | 47,902 | <0.001 | <0.001 | 0.014 | 0.013 | 1.171 | No | No |
| Absolute weekday–weekend midpoint difference | Suicidal ideation | 48,416 | 47,902 | <0.001 | <0.001 | 0.217 | 0.040 | 1.304 | No | Yes |
| Absolute weekday–weekend midpoint difference | Suicide plan | 48,416 | 47,902 | <0.001 | <0.001 | 0.181 | 0.086 | 1.627 | No | No |
| Absolute weekday–weekend midpoint difference | Suicide attempt | 48,416 | 47,902 | <0.001 | <0.001 | 0.501 | 0.936 | 2.138 | No | No |

Panel B compares the locked primary spline models (*N* = 48,416) with models excluding 514 observations with extreme extended-scale MSFsc values below 24 or above 36 hours (restricted *N* = 47,902). The same knots, reference values, covariates, and prediction grids were retained. This restriction was used as a post hoc sensitivity analysis and was not treated as an official KYRBS exclusion rule.

**Panel C. Additional adjustment for academic-load proxy**

| **Exposure** | **Outcome** | **Same-sample *N*** | **Baseline overall *p*** | **Plus proxy overall *p*** | **Baseline nonlinearity *p*** | **Plus proxy nonlinearity *p*** | **Maximum OR-ratio change** | **Overall significance changed** | **Nonlinearity significance changed** |
| --- | --- | --- | --- | --- | --- | --- | --- | --- | --- |
| MSFsc | Depressive mood | 47,565 | <0.001 | <0.001 | 0.020 | 0.018 | 1.003 | No | No |
| MSFsc | Suicidal ideation | 47,565 | 0.001 | 0.002 | 0.007 | 0.006 | 1.011 | No | No |
| MSFsc | Suicide plan | 47,565 | <0.001 | <0.001 | <0.001 | <0.001 | 1.005 | No | No |
| MSFsc | Suicide attempt | 47,565 | 0.034 | 0.025 | 0.990 | 0.983 | 1.014 | No | No |
| Weekly average sleep duration | Depressive mood | 47,565 | <0.001 | <0.001 | <0.001 | <0.001 | 1.013 | No | No |
| Weekly average sleep duration | Suicidal ideation | 47,565 | <0.001 | <0.001 | <0.001 | <0.001 | 1.021 | No | No |
| Weekly average sleep duration | Suicide plan | 47,565 | <0.001 | <0.001 | <0.001 | <0.001 | 1.013 | No | No |
| Weekly average sleep duration | Suicide attempt | 47,565 | <0.001 | <0.001 | <0.001 | <0.001 | 1.021 | No | No |
| Absolute weekday–weekend midpoint difference | Depressive mood | 47,565 | <0.001 | <0.001 | 0.017 | 0.020 | 1.004 | No | No |
| Absolute weekday–weekend midpoint difference | Suicidal ideation | 47,565 | <0.001 | <0.001 | 0.218 | 0.223 | 1.007 | No | No |
| Absolute weekday–weekend midpoint difference | Suicide plan | 47,565 | <0.001 | <0.001 | 0.180 | 0.204 | 1.006 | No | No |
| Absolute weekday–weekend midpoint difference | Suicide attempt | 47,565 | <0.001 | <0.001 | 0.576 | 0.584 | 1.012 | No | No |

Panel C compares same-sample baseline spline models with models additionally adjusted for a restricted cubic spline of weekly academic-purpose sitting time (*N* = 47,565). The proxy combines school and private-academy classes, homework, and studying and does not isolate hagwon attendance or duration.

Overall and nonlinearity significance changes indicate whether the corresponding *p* value crossed the 0.05 threshold. These analyses assess robustness and should not be interpreted as proving causal independence or mediation. Signed midpoint-difference, full weekend-bedtime exclusion, prespecified plausible-range, and weekday/weekend sleep-duration analyses are reported separately.

**Supplementary Table S6. Detailed sensitivity analyses using signed midpoint difference and restrictions for potentially implausible sleep-timing responses**

**Panel A. Signed weekend-minus-weekday midpoint difference**

| **Outcome** | ***N*** | **Evening vs Morning** | **Intermediate vs Morning** | **Sleep-timing group omnibus *p*** | **Continuous MSFsc, per 1 h** | **Signed midpoint difference, per 1 h** |
| --- | --- | --- | --- | --- | --- | --- |
| Depressive mood | 48,416 | 0.988 (0.901–1.085) | 0.876 (0.822–0.933) | <0.001 | 0.887 (0.862–0.912) | 1.146 (1.114–1.178) |
| Suicidal ideation | 48,416 | 0.981 (0.857–1.124) | 0.838 (0.764–0.919) | <0.001 | 0.918 (0.882–0.956) | 1.122 (1.077–1.170) |
| Suicide plan | 48,416 | 0.774 (0.635–0.945) | 0.653 (0.573–0.745) | <0.001 | 0.827 (0.778–0.878) | 1.227 (1.153–1.305) |
| Suicide attempt | 48,416 | 0.663 (0.506–0.868) | 0.684 (0.577–0.811) | <0.001 | 0.824 (0.763–0.891) | 1.246 (1.149–1.351) |

Panel A replaces the absolute weekday-weekend midpoint difference with the signed weekend-minus-weekday midpoint difference, for which positive values indicate a later weekend midpoint. The analytic sample was the final complete-case sample (*N* = 48,416). Because MSFsc and the signed midpoint difference are derived from overlapping sleep-timing components, their mutually adjusted estimates are conditional and may be influenced by mathematical coupling. These estimates should not be interpreted as independent causal effects or as evidence that later sleep timing is protective.

**Panel B. Exclusion of weekend bedtime responses from 04:00 to 19:59**

| **Outcome** | ***N*** | **Evening vs Morning** | **Intermediate vs Morning** | **Sleep-timing group omnibus *p*** | **Continuous MSFsc, per 1 h** | **Absolute midpoint difference, per 1 h** |
| --- | --- | --- | --- | --- | --- | --- |
| Depressive mood | 43,951 | 0.873 (0.787–0.969) | 0.867 (0.814–0.924) | <0.001 | 0.943 (0.916–0.970) | 1.115 (1.084–1.148) |
| Suicidal ideation | 43,951 | 0.887 (0.762–1.032) | 0.850 (0.774–0.933) | 0.002 | 0.932 (0.897–0.968) | 1.102 (1.058–1.147) |
| Suicide plan | 43,951 | 0.684 (0.547–0.856) | 0.669 (0.584–0.766) | <0.001 | 0.837 (0.794–0.883) | 1.169 (1.099–1.243) |
| Suicide attempt | 43,951 | 0.571 (0.425–0.767) | 0.655 (0.551–0.780) | <0.001 | 0.848 (0.794–0.907) | 1.239 (1.156–1.329) |

Panel B excludes participants whose reported weekend bedtime fell between 04:00 and 19:59, using the weekend-bedtime sensitivity restriction. Within the final complete-case sample, 4,465 participants met this criterion and were excluded, leaving 43,951 participants. The corresponding number among all 48,426 participants with valid sleep-derived variables was 4,467; the difference of two participants reflects the final complete-case restriction. Models otherwise retained the primary adjustment set.

**Panel C. Restriction to 3-14 hours of weekday and weekend sleep**

| **Outcome** | ***N*** | **Evening vs Morning** | **Intermediate vs Morning** | **Sleep-timing group omnibus *p*** | **Continuous MSFsc, per 1 h** | **Absolute midpoint difference, per 1 h** |
| --- | --- | --- | --- | --- | --- | --- |
| Depressive mood | 46,993 | 0.958 (0.871–1.053) | 0.897 (0.841–0.956) | <0.001 | 0.970 (0.945–0.996) | 1.075 (1.045–1.105) |
| Suicidal ideation | 46,993 | 0.992 (0.864–1.140) | 0.863 (0.785–0.949) | <0.001 | 0.988 (0.955–1.021) | 1.074 (1.034–1.114) |
| Suicide plan | 46,993 | 0.815 (0.666–0.997) | 0.683 (0.596–0.783) | <0.001 | 0.930 (0.881–0.982) | 1.120 (1.056–1.188) |
| Suicide attempt | 46,993 | 0.696 (0.538–0.900) | 0.713 (0.600–0.846) | <0.001 | 0.926 (0.885–0.969) | 1.143 (1.083–1.205) |

Panel C restricts both weekday and weekend derived sleep durations to the prespecified broad plausible range of 3-14 hours, inclusive. This restriction excluded 1,423 participants from the final complete-case sample and retained 46,993. Models otherwise retain the primary adjustment set.

Values are odds ratios (95% confidence intervals) from survey-weighted logistic regression models. Categorical models used the morning group as the reference category. Continuous models estimated the association per 1-hour increase in MSFsc and in the indicated midpoint-difference measure. All models adjusted for sex, grade, categorical perceived socioeconomic status, categorical academic performance, and weekly average sleep duration. Models in Panels B and C additionally included the absolute weekday-weekend midpoint difference. The same analytic sample was used across all four outcomes within each panel. These analyses should be interpreted as sensitivity analyses rather than alternative primary models.

**Supplementary Table S7. Associations in models separating weekday sleep duration, weekend sleep duration, and weekend sleep extension**

**Panel A. Weekday sleep-duration models**

| **Outcome** | ***N*** | **Evening vs Morning** | **Intermediate vs Morning** | **Sleep-timing group omnibus *p*** | **Continuous MSFsc, per 1 h** | **Weekday sleep, per 1 h** |
| --- | --- | --- | --- | --- | --- | --- |
| Depressive mood | 48,416 | 1.008 (0.928–1.094) | 0.903 (0.853–0.957) | <0.001 | 0.988 (0.974–1.003) | 0.815 (0.800–0.829) |
| Suicidal ideation | 48,416 | 1.137 (1.008–1.281) | 0.914 (0.840–0.995) | <0.001 | 1.022 (1.000–1.044) | 0.794 (0.775–0.814) |
| Suicide plan | 48,416 | 0.910 (0.761–1.088) | 0.727 (0.641–0.826) | <0.001 | 0.984 (0.954–1.015) | 0.790 (0.755–0.826) |
| Suicide attempt | 48,416 | 0.871 (0.697–1.087) | 0.804 (0.684–0.945) | 0.025 | 0.998 (0.965–1.032) | 0.786 (0.749–0.825) |

Panel A replaces weekly average sleep duration with weekday sleep duration. Models also include the absolute weekday-weekend sleep midpoint difference.

**Panel B. Weekend sleep-duration models**

| **Outcome** | ***N*** | **Evening vs Morning** | **Intermediate vs Morning** | **Sleep-timing group omnibus *p*** | **Continuous MSFsc, per 1 h** | **Weekend sleep, per 1 h** |
| --- | --- | --- | --- | --- | --- | --- |
| Depressive mood | 48,416 | 1.114 (1.021–1.215) | 0.960 (0.904–1.019) | <0.001 | 0.982 (0.963–1.001) | 0.951 (0.938–0.963) |
| Suicidal ideation | 48,416 | 1.133 (1.002–1.283) | 0.928 (0.851–1.012) | <0.001 | 0.996 (0.971–1.021) | 0.913 (0.898–0.929) |
| Suicide plan | 48,416 | 0.893 (0.751–1.061) | 0.734 (0.647–0.831) | <0.001 | 0.961 (0.930–0.993) | 0.899 (0.875–0.924) |
| Suicide attempt | 48,416 | 0.855 (0.688–1.064) | 0.813 (0.691–0.956) | 0.044 | 0.978 (0.946–1.011) | 0.894 (0.865–0.925) |

Panel B replaces weekly average sleep duration with weekend sleep duration. Models also include the absolute weekday-weekend sleep midpoint difference.

**Panel C. Weekday sleep duration and weekend sleep-extension models**

| **Outcome** | ***N*** | **Evening vs Morning** | **Intermediate vs Morning** | **Sleep-timing group omnibus *p*** | **Continuous MSFsc, per 1 h** | **Weekday sleep, per 1 h** | **Weekend extension, per 1 h** |
| --- | --- | --- | --- | --- | --- | --- | --- |
| Depressive mood | 48,416 | 0.976 (0.893–1.067) | 0.890 (0.838–0.946) | <0.001 | 0.975 (0.958–0.992) | 0.805 (0.790–0.821) | 0.982 (0.969–0.995) |
| Suicidal ideation | 48,416 | 0.972 (0.858–1.102) | 0.851 (0.780–0.929) | <0.001 | 0.982 (0.961–1.003) | 0.766 (0.746–0.786) | 0.944 (0.928–0.961) |
| Suicide plan | 48,416 | 0.752 (0.631–0.896) | 0.666 (0.587–0.756) | <0.001 | 0.947 (0.919–0.975) | 0.755 (0.721–0.791) | 0.927 (0.902–0.953) |
| Suicide attempt | 48,416 | 0.711 (0.570–0.887) | 0.733 (0.623–0.862) | <0.001 | 0.960 (0.931–0.989) | 0.749 (0.712–0.788) | 0.922 (0.892–0.953) |

Panel C includes weekday sleep duration and weekend sleep extension, defined as weekend minus weekday sleep duration, together with the absolute weekday-weekend sleep midpoint difference.

Values are odds ratios (95% confidence intervals) from survey-weighted logistic regression models. Categorical models used the morning group as the reference category. Continuous models estimated associations per 1-hour increase in MSFsc and in the displayed sleep-duration measure. Sleep-duration coefficients are reported from the continuous-MSFsc models. All models adjusted for sex, grade, categorical perceived socioeconomic status, and categorical academic performance. All analyses used the same final complete-case sample (*N* = 48,416). Because MSFsc, sleep duration, weekend sleep extension, and midpoint difference share component sleep-timing variables, mutually adjusted estimates are conditional and may be affected by mathematical coupling; they should not be interpreted as independent causal effects or mediation estimates.

**Supplementary Table S8. Direct MSFsc-based sleep-timing group contrasts within sleep-duration strata with Benjamini–Hochberg false-discovery-rate adjustment**

**Panel A. Depressive mood**

| **Sleep-duration stratum** | **Contrast** | **OR (95% CI)** | **Raw *p*** | **BH-FDR *q*** | ***q* < 0.05** |
| --- | --- | --- | --- | --- | --- |
| <5 h | Evening vs Morning | 0.794 (0.609–1.037) | 0.090 | 0.309 | No |
|  | Intermediate vs Morning | **0.652 (0.518–0.820)** | **<0.001** | **0.006** | **Yes** |
|  | Evening vs Intermediate | 1.219 (1.032–1.440) | 0.020 | 0.178 | No |
| 5–<6 h | Evening vs Morning | 0.962 (0.793–1.168) | 0.697 | 0.838 | No |
|  | Intermediate vs Morning | 0.877 (0.750–1.025) | 0.098 | 0.321 | No |
|  | Evening vs Intermediate | 1.098 (0.960–1.256) | 0.173 | 0.401 | No |
| 6–<7 h | Evening vs Morning | 1.004 (0.841–1.200) | 0.962 | 0.976 | No |
|  | Intermediate vs Morning | 0.937 (0.828–1.061) | 0.305 | 0.610 | No |
|  | Evening vs Intermediate | 1.072 (0.942–1.219) | 0.292 | 0.601 | No |
| 7–<8 h | Evening vs Morning | 1.035 (0.843–1.272) | 0.740 | 0.838 | No |
|  | Intermediate vs Morning | 0.892 (0.786–1.011) | 0.074 | 0.303 | No |
|  | Evening vs Intermediate | 1.161 (0.996–1.355) | 0.057 | 0.279 | No |
| 8–<9 h | Evening vs Morning | 0.738 (0.549–0.991) | 0.043 | 0.279 | No |
|  | Intermediate vs Morning | **0.754 (0.654–0.869)** | **<0.001** | **0.004** | **Yes** |
|  | Evening vs Intermediate | 0.979 (0.763–1.255) | 0.866 | 0.931 | No |
| ≥9 h | Evening vs Morning | 1.023 (0.684–1.532) | 0.910 | 0.957 | No |
|  | Intermediate vs Morning | 1.043 (0.847–1.284) | 0.694 | 0.838 | No |
|  | Evening vs Intermediate | 0.982 (0.692–1.393) | 0.917 | 0.957 | No |

**Panel B. Suicidal ideation**

| **Sleep-duration stratum** | **Contrast** | **OR (95% CI)** | **Raw *p*** | **BH-FDR *q*** | ***q* < 0.05** |
| --- | --- | --- | --- | --- | --- |
| <5 h | Evening vs Morning | 1.060 (0.765–1.470) | 0.724 | 0.838 | No |
|  | Intermediate vs Morning | 0.880 (0.673–1.152) | 0.353 | 0.669 | No |
|  | Evening vs Intermediate | 1.205 (0.977–1.486) | 0.082 | 0.309 | No |
| 5–<6 h | Evening vs Morning | 1.065 (0.808–1.405) | 0.655 | 0.838 | No |
|  | Intermediate vs Morning | 0.846 (0.686–1.044) | 0.119 | 0.344 | No |
|  | Evening vs Intermediate | 1.259 (1.046–1.516) | 0.015 | 0.156 | No |
| 6–<7 h | Evening vs Morning | 0.946 (0.748–1.197) | 0.645 | 0.838 | No |
|  | Intermediate vs Morning | 0.871 (0.743–1.020) | 0.087 | 0.309 | No |
|  | Evening vs Intermediate | 1.087 (0.918–1.287) | 0.335 | 0.651 | No |
| 7–<8 h | Evening vs Morning | 1.070 (0.802–1.427) | 0.645 | 0.838 | No |
|  | Intermediate vs Morning | 0.765 (0.638–0.916) | 0.004 | 0.053 | No |
|  | Evening vs Intermediate | **1.399 (1.126–1.739)** | **0.002** | **0.044** | **Yes** |
| 8–<9 h | Evening vs Morning | 0.853 (0.569–1.279) | 0.441 | 0.775 | No |
|  | Intermediate vs Morning | 0.964 (0.779–1.191) | 0.731 | 0.838 | No |
|  | Evening vs Intermediate | 0.885 (0.638–1.229) | 0.466 | 0.779 | No |
| ≥9 h | Evening vs Morning | 0.807 (0.427–1.526) | 0.509 | 0.779 | No |
|  | Intermediate vs Morning | 0.970 (0.725–1.297) | 0.835 | 0.911 | No |
|  | Evening vs Intermediate | 0.832 (0.484–1.432) | 0.507 | 0.779 | No |

**Panel C. Suicide plan**

| **Sleep-duration stratum** | **Contrast** | **OR (95% CI)** | **Raw *p*** | **BH-FDR *q*** | ***q* < 0.05** |
| --- | --- | --- | --- | --- | --- |
| <5 h | Evening vs Morning | 0.770 (0.519–1.142) | 0.193 | 0.435 | No |
|  | Intermediate vs Morning | 0.597 (0.409–0.870) | 0.007 | 0.089 | No |
|  | Evening vs Intermediate | 1.290 (0.976–1.705) | 0.073 | 0.303 | No |
| 5–<6 h | Evening vs Morning | 0.982 (0.649–1.486) | 0.932 | 0.959 | No |
|  | Intermediate vs Morning | 0.889 (0.642–1.230) | 0.477 | 0.779 | No |
|  | Evening vs Intermediate | 1.105 (0.832–1.466) | 0.489 | 0.779 | No |
| 6–<7 h | Evening vs Morning | 1.052 (0.725–1.526) | 0.789 | 0.874 | No |
|  | Intermediate vs Morning | 0.817 (0.624–1.071) | 0.143 | 0.369 | No |
|  | Evening vs Intermediate | 1.287 (0.995–1.665) | 0.054 | 0.279 | No |
| 7–<8 h | Evening vs Morning | 0.585 (0.336–1.019) | 0.058 | 0.279 | No |
|  | Intermediate vs Morning | **0.523 (0.385–0.709)** | **<0.001** | **0.002** | **Yes** |
|  | Evening vs Intermediate | 1.120 (0.744–1.685) | 0.586 | 0.828 | No |
| 8–<9 h | Evening vs Morning | 0.602 (0.313–1.155) | 0.127 | 0.351 | No |
|  | Intermediate vs Morning | 0.733 (0.532–1.011) | 0.058 | 0.279 | No |
|  | Evening vs Intermediate | 0.821 (0.461–1.461) | 0.502 | 0.779 | No |
| ≥9 h | Evening vs Morning | 0.829 (0.352–1.952) | 0.668 | 0.838 | No |
|  | Intermediate vs Morning | 0.721 (0.455–1.143) | 0.164 | 0.394 | No |
|  | Evening vs Intermediate | 1.150 (0.495–2.672) | 0.745 | 0.838 | No |

**Panel D. Suicide attempt**

| **Sleep-duration stratum** | **Contrast** | **OR (95% CI)** | **Raw *p*** | **BH-FDR *q*** | ***q* < 0.05** |
| --- | --- | --- | --- | --- | --- |
| <5 h | Evening vs Morning | 0.706 (0.433–1.152) | 0.163 | 0.394 | No |
|  | Intermediate vs Morning | 0.704 (0.440–1.124) | 0.141 | 0.369 | No |
|  | Evening vs Intermediate | 1.003 (0.690–1.458) | 0.986 | 0.986 | No |
| 5–<6 h | Evening vs Morning | 0.750 (0.454–1.239) | 0.261 | 0.552 | No |
|  | Intermediate vs Morning | 0.829 (0.550–1.250) | 0.370 | 0.683 | No |
|  | Evening vs Intermediate | 0.905 (0.654–1.252) | 0.545 | 0.806 | No |
| 6–<7 h | Evening vs Morning | 1.114 (0.623–1.990) | 0.715 | 0.838 | No |
|  | Intermediate vs Morning | 0.931 (0.627–1.380) | 0.720 | 0.838 | No |
|  | Evening vs Intermediate | 1.197 (0.780–1.836) | 0.410 | 0.737 | No |
| 7–<8 h | Evening vs Morning | 0.835 (0.456–1.531) | 0.560 | 0.806 | No |
|  | Intermediate vs Morning | 0.738 (0.503–1.082) | 0.120 | 0.344 | No |
|  | Evening vs Intermediate | 1.132 (0.702–1.826) | 0.610 | 0.838 | No |
| 8–<9 h | Evening vs Morning | 0.447 (0.220–0.908) | 0.026 | 0.209 | No |
|  | Intermediate vs Morning | 0.763 (0.504–1.154) | 0.199 | 0.435 | No |
|  | Evening vs Intermediate | 0.586 (0.306–1.121) | 0.106 | 0.333 | No |
| ≥9 h | Evening vs Morning | 1.390 (0.467–4.138) | 0.554 | 0.806 | No |
|  | Intermediate vs Morning | 0.559 (0.310–1.009) | 0.053 | 0.279 | No |
|  | Evening vs Intermediate | 2.485 (0.910–6.787) | 0.076 | 0.303 | No |

Values are odds ratios (95% confidence intervals) for the first sleep-timing group named relative to the second sleep-timing group named within the same sleep-duration stratum. Each sleep-duration stratum was analyzed in a separate survey-weighted logistic regression model using the KYRBS complex-sample design and adjusting for sex, grade, categorical perceived socioeconomic status, categorical academic performance, and absolute weekday–weekend sleep midpoint difference. Raw *p* values are shown together with Benjamini–Hochberg false-discovery-rate adjusted *q* values calculated across all 72 pairwise contrasts (six sleep-duration strata × three sleep-timing group contrasts × four outcomes). Rows with *q* < 0.05 are bolded. These joint-category analyses are exploratory. Because Supplementary Table S9 uses a single pooled 18-level joint-profile model with common covariate coefficients across strata, the estimates in Supplementary Tables S8 and S9 are not expected to be algebraically identical. Direct pairwise contrasts should be interpreted together with confidence intervals and the omnibus sleep-timing group-by-sleep-duration interaction tests; differences between separate point estimates do not themselves establish a statistically significant between-sleep-timing group contrast.

**Supplementary Table S9. Exploratory joint associations of MSFsc-based sleep-timing group and sleep-duration category with mental health outcomes**

**Panel A. Morning**

| **Sleep duration** | **Depressive mood aOR (95% CI)** | **Suicidal ideation aOR (95% CI)** | **Suicide plan aOR (95% CI)** | **Suicide attempt aOR (95% CI)** |
| --- | --- | --- | --- | --- |
| <5 h | 3.13 (2.46–3.98)*** | 3.39 (2.52–4.57)*** | 4.17 (2.81–6.17)*** | 4.50 (2.65–7.62)*** |
| 5 to <6 h | 1.93 (1.63–2.29)*** | 2.17 (1.72–2.75)*** | 1.80 (1.22–2.65)** | 2.37 (1.43–3.94)*** |
| 6 to <7 h | 1.44 (1.25–1.66)*** | 1.77 (1.45–2.16)*** | 1.42 (1.01–2.00)* | 1.36 (0.84–2.18) |
| 7 to <8 h | 1.16 (1.01–1.32)* | 1.33 (1.08–1.63)** | 1.30 (0.94–1.78) | 1.36 (0.87–2.11) |
| 8 to <9 h | 1.00 (Reference) | 1.00 (Reference) | 1.00 (Reference) | 1.00 (Reference) |
| ≥9 h | 0.91 (0.78–1.07) | 1.04 (0.82–1.32) | 1.18 (0.83–1.68) | 1.40 (0.92–2.14) |

**Panel B. Intermediate**

| **Sleep duration** | **Depressive mood aOR (95% CI)** | **Suicidal ideation aOR (95% CI)** | **Suicide plan aOR (95% CI)** | **Suicide attempt aOR (95% CI)** |
| --- | --- | --- | --- | --- |
| <5 h | 2.03 (1.78–2.32)*** | 2.95 (2.43–3.58)*** | 2.39 (1.77–3.23)*** | 3.09 (2.12–4.50)*** |
| 5 to <6 h | 1.62 (1.44–1.82)*** | 1.80 (1.51–2.15)*** | 1.56 (1.19–2.05)** | 1.97 (1.39–2.78)*** |
| 6 to <7 h | 1.32 (1.18–1.47)*** | 1.52 (1.28–1.81)*** | 1.07 (0.81–1.41) | 1.20 (0.83–1.72) |
| 7 to <8 h | 1.05 (0.94–1.17) | 1.02 (0.85–1.23) | 0.76 (0.58–1.00)* | 0.97 (0.68–1.40) |
| 8 to <9 h | 0.82 (0.72–0.93)** | 1.03 (0.84–1.26) | 0.79 (0.58–1.09) | 0.92 (0.61–1.40) |
| ≥9 h | 0.97 (0.81–1.18) | 1.02 (0.79–1.34) | 0.74 (0.48–1.14) | 0.64 (0.36–1.14) |

**Panel C. Evening**

| **Sleep duration** | **Depressive mood aOR (95% CI)** | **Suicidal ideation aOR (95% CI)** | **Suicide plan aOR (95% CI)** | **Suicide attempt aOR (95% CI)** |
| --- | --- | --- | --- | --- |
| <5 h | 2.42 (2.07–2.82)*** | 3.44 (2.77–4.26)*** | 3.22 (2.35–4.40)*** | 3.02 (1.96–4.64)*** |
| 5 to <6 h | 1.59 (1.38–1.84)*** | 2.18 (1.78–2.68)*** | 1.64 (1.18–2.28)** | 1.82 (1.20–2.77)** |
| 6 to <7 h | 1.35 (1.18–1.55)*** | 1.61 (1.31–1.97)*** | 1.18 (0.87–1.60) | 1.28 (0.85–1.93) |
| 7 to <8 h | 1.25 (1.07–1.46)** | 1.45 (1.14–1.83)** | 1.05 (0.74–1.51) | 1.05 (0.65–1.69) |
| 8 to <9 h | 1.00 (0.82–1.23) | 1.12 (0.83–1.50) | 0.85 (0.54–1.33) | 1.04 (0.59–1.84) |
| ≥9 h | 1.09 (0.82–1.45) | 0.86 (0.55–1.36) | 0.62 (0.30–1.26) | 0.91 (0.38–2.14) |

Values are adjusted odds ratios from a single survey-weighted logistic regression model for each outcome using the Morning group with 8 to <9 h of sleep as the reference profile. The pooled 18-level joint-profile model estimated common covariate coefficients across all sleep-duration strata and adjusted for sex, school grade, five-level categorical perceived socioeconomic status, five-level categorical academic performance, and the absolute weekday–weekend sleep midpoint difference. All models used the final complete-case sample (*N* = 48,416). These joint-profile analyses are exploratory. Supplementary Table S8 reports direct sleep-timing group contrasts estimated from separate regression models within each sleep-duration stratum; therefore, those contrast estimates are not algebraic transformations of the pooled joint-profile coefficients shown here. aOR, adjusted odds ratio; CI, confidence interval; MSFsc, corrected midpoint of sleep on free days. * *p* < 0.05; ** *p* < 0.01; *** *p* < 0.001.

**Supplementary Figure S1. Survey-weighted distribution of corrected mid-sleep on free days and classification thresholds.** The curve shows the survey-weighted density distribution of corrected mid-sleep on free days (MSFsc) among 48,426 participants with valid sleep-derived variables. Vertical dashed lines indicate the locked unweighted 20th- and 80th-percentile thresholds of 27.7619 and 29.9524 extended hours, corresponding to approximately 03:46 and 05:57, respectively. The labeled regions indicate the morning, intermediate, and evening MSFsc-based sleep-timing groups. The displayed x-axis was restricted to the central distribution for visual clarity; full distributional statistics and classification rules are presented in Supplementary Table S2.

**Supplementary Figure S2. Survey-weighted distributions of weekly average sleep duration and the absolute weekday–weekend sleep midpoint difference according to MSFsc-based sleep-timing group.** Panel A shows the survey-weighted density distributions of weekly average sleep duration, and Panel B shows the survey-weighted density distributions of the absolute weekday–weekend sleep midpoint difference. Curves are presented separately for the morning, intermediate, and evening MSFsc-based sleep-timing groups. The analytic sample included 48,426 participants with valid sleep-derived variables.
